# Unravelling the physiological roles of *mazEF* toxin–antitoxin system on clinical MRSA strain by CRISPR RNA-guided cytidine deaminase

**DOI:** 10.1186/s12929-022-00810-5

**Published:** 2022-05-07

**Authors:** Sonia Jain, Arghya Bhowmick, Bohyun Jeong, Taeok Bae, Abhrajyoti Ghosh

**Affiliations:** 1 Infectious Disease and Immunology Division, CSIR-Indian Institute of Chemical Biology, Kolkata, 700032 India; 2 Department of Biochemistry, Bose Institute, EN Block, Sector-V, Kolkata, 700091 India; 3 Department of Microbiology, Kosin University College of Medicine, Busan, 49267 South Korea; 4 Department of Microbiology and Immunology, Indiana University, School of Medicine-Northwest, Gary, IN 46408-1197 USA

**Keywords:** MRSA, Biofilm, Antibiotic resistance, Virulence, CRISPR–Cas9, Toxin–antitoxin

## Abstract

**Background:**

Curiosity on toxin–antitoxin modules has increased intensely over recent years as it is ubiquitously present in many bacterial genomes, including pathogens like Methicillin-resistant *Staphylococcus aureus* (MRSA). Several cellular functions of TA systems have been proposed however, their exact role in cellular physiology remains unresolved.

**Methods:**

This study aims to find out the impact of the *mazEF* toxin–antitoxin module on biofilm formation, pathogenesis, and antibiotic resistance in an isolated clinical ST239 MRSA strain, by constructing *mazE* and *mazF* mutants using CRISPR–cas9 base-editing plasmid (pnCasSA-BEC). Transcriptome analysis (RNA-seq) was performed for the *mazE* antitoxin mutant in order to identify the differentially regulated genes. The biofilm formation was also assessed for the mutant strains. Antibiogram profiling was carried out for both the generated mutants followed by murine experiment to determine the pathogenicity of the constructed strains.

**Results:**

For the first time our work showed, that MazF promotes *cidA* mediated cell death and lysis for biofilm formation without playing any significant role in host virulence as suggested by the murine experiment. Interestingly, the susceptibility to oxacillin, daptomycin and vancomycin was reduced significantly by the activated MazF toxin in the *mazE* mutant strain.

**Conclusions:**

Our study reveals that activated MazF toxin leads to resistance to antibiotics like oxacillin, daptomycin and vancomycin. Therefore, in the future, any potential antibacterial drug can be designed to target MazF toxin against the problematic multi-drug resistant bug.

**Supplementary Information:**

The online version contains supplementary material available at 10.1186/s12929-022-00810-5.

## Introduction

A commensal bacterium, *Staphylococcus aureus*, is the most common pathogen involved in several nosocomial chronic infections [1, 2]. Some strains of *S. aureus* are genetically distinct in their ability to resist antibiotics (mainly beta-lactams), represented as Methicillin-resistant *Staphylococcus aureus* (MRSA) [3]._._ MRSA had been registered by the U.S. Centers for Disease Control and Prevention (CDC) as a “superbug” owing to its multidrug-resistant phenotype. The infection caused by these opportunistic pathogens is often ascribed to implanted medical devices, where their characteristic biofilm formation renders them resistant to different antibiotics and immune cells, thus aggravating the condition of the post-operative patients [4, 5]. Although the mechanism behind biofilm formation and its relation to multidrug tolerance-related pathogenicity in MRSAs are not well characterized, many researchers speculate that the chromosomally encoded toxin–antitoxin system present in MRSA might play an important role in this regard [6, 7]. In *S. aureus* strains, the *mazEF* toxin–antitoxin module (where *mazF* encodes the toxin protein while *mazE* encodes the labile antitoxin) has been extensively studied [8]. *S. aureus* MazF toxin has also been shown to act as an endoribonuclease that inhibits translation by cleaving single-stranded mRNA at a specific site [8]. The MazF toxin overexpression in *S. aureus* cells targets the essential cellular and metabolic processes, resulting in either a dormant state of cells known as persisters or may lead to cell death [9–12]. In summary, the bacteria respond to antibiotic exposure by activating intracellular toxin proteins, which halt cellular growth and induce the formation of persisters cells that survives the antibiotic insult [7, 9]. After the antibiotic exposure ceases, persisters can regrow by synthesizing antitoxin proteins that sequester toxin proteins and restores cell growth [6, 7]. The motive behind this study was to find out the role of the *mazEF* toxin–antitoxin module in clinically isolated MRSA, which are genetically distinct from common lab strains. The corresponding strain was isolated from a patient’s pus sample, admitted to a tertiary care hospital, Kolkata (India), due to post-operative infection. From our previous finding, the epidemiological characterization of this hospital-acquired (HA)-MRSA strain shows that it belongs to ST239, *SCC mec III*, *agr* system I, containing exotoxins [13]. We successfully generated *mazE* and *mazF* mutants using the CRISPR system and performed RNA-sequencing of the *mazE* mutant strain, to identify the differentially expressed genes. This study helped in the better characterization of the *mazEF* TA system. The use of the pnCasSA-BEC system enables efficient point mutations and gene inactivation in the genomes of these directly isolated MRSA strains having low transformation efficiency. Compared to the older traditional method of plasmid delivery systems (like the use of pKOR1, pMad, pIMAY, improved DC10B host, IMxxB strains of different clonal complexes) which was labor-intensive and time-consuming, pnCasSA-BEC provides simple, rapid, sequence-specific point mutation [14–18]. Previous studies have relied on overexpressing the MazF toxin protein by increasing the toxin:antitoxin ratio to check the effect of the toxin on the cells, which led to an abnormal abundance of the toxin protein leading to erroneous results [19]. On the contrary, in this study, the natural activation of MazF toxin and MazE antitoxin better resembled the cellular physiological condition.

## Materials and methods

### Ethics statement

The animal study was executed by following the Guide for the Care and Use of Laboratory Animals of the National Institutes of Health. The followed animal study protocol was approved by the Committee on the Ethics of Animal Experiments of the Indiana University School of Medicine-Northwest (Protocol Number: NW-48). Every possible effort was made to lessen the suffering of the animals.

### Strains, primers and mutational strategy using CRISPR plasmid

All the strains used and primers designed for this study are enlisted in (Additional file 1: Table S1). We used a clinical MRSA strain, namely P-1780 (belonging to ST239), isolated from a patient’s pus, from our previous study [13]. The *mazF* and *mazE* mutants were constructed using the CRISPR-based RNA-guided cytidine deaminase (pnCasSA-BEC) system [17]. The plasmid system comprising Cas9 nickase (Cas9D10A) attached to cytidine deaminase (APOBEC1) was directed to *mazE* and *mazF* genomic locus for inducing point mutation in those genes [17]. This point mutation generates a premature stop codon leading to *mazE* and *mazF* mutants respectively [17]. The pnCasSA-BEC plasmid system generates nicks on the unedited gDNA strand and directly mediates the conversion of (C) cytidine to (U) uridine while relying on DNA replication to accomplish C/T (G/A) base conversion without the repair by donor templates [17]. The sequences of *mazEF* gene of *S. aureus* were obtained from NCBI (USA300_FPR3757). All the possible PAM (NGG) sites present were scanned through the entire *mazEF* genome. 20 bp-spacer sequences with editable sites and the occurrence of C(s) at the 14 bp upstream of PAM (NGG) site in the *mazE* and *mazF* locus were selected. Bioinformatic scanning using Circos application was carried out, for analyzing editable sites of the pnCasSA-BEC system in the genome of the MRSA ST239 strain [20]. Then the annealed phosphorylated spacer was fused with the pnCasSA-BEC plasmid using a golden gate assembly reaction. The golden gate assembly product was then transformed into competent *E.coli* DH5α cells. The required constructed pnCasSA-BEC-*mazE/mazF* spacer plasmid was verified by PCR, followed by sequencing. The site that introduced stop codons after the C/T conversion (UAG for *mazF* and UAA for *mazE*) were treated as editable stop sites with the help of the Integrative Genomics Viewer [21].

### Base editing in RN4220 strain

Next, the assembled pnCasSA-BEC-*mazE*/*mazF*sp plasmids were individually transformed into the laboratory RN4220 *S. aureus* competent cells by electroporation (details in Additional file 1).

After transformation, the genomic DNAs from the colonies were extracted using modified short phenol chloroform method [22]. DNA sequence covering the target site was amplified by PCR using primers listed in Additional file 1: Table S1. The successful mutation in the target site was further confirmed by Sanger’s sequencing as described in previous studies [14, 17]

### Base editing in clinical ST239 MRSA strain

Plasmid transformation efficiency by electroporation in the ST239 strain is very low; therefore, we used the phage transduction method (details in Additional file 1) to transduce the pnCasSA-BEC *mazF*sp*/mazE*sp plasmid with the desired mutation into the wildtype P-1780 strain from RN4220 strain. The plasmids were simultaneously transduced into the strain P-1780 using the phage phi85 and the resulting cells of *mazE* and *mazF* mutants (confirmed by Sanger’s sequencing) were plated on a TSB agar plate comprising 10 μg/ml of chloramphenicol at 30 °C. The number of colonies in each of the plates was counted as CFU/ml to confirm the effect of MazF toxin in the *mazE* mutant. The wildtype as well as the *mazE and mazF* mutant MRSA cells were suspended in Brain heart infusion (BHI) (Becton Dickinson) containing 10% glycerol for long-term storage and kept at − 80 °C. The frozen culture was thawed at room temperature before using it for any further experiments as described previously [14, 17].

### Complement strain generation

To complement the mutations, the complete *mazE and mazF* gene from P-1780 strain were individually PCR amplified from 237 and 158 bps upstream of their open reading frame respectively and cloned into a single copy plasmid vector pCL55 using *EcoRI* and *BamHI* restriction site [23]. The upstream region of the ORF contained the promoter and the ribosome binding sites responsible for protein synthesis. The plasmid was transduced into the mutant strains (by the same method mentioned above) and selected with 5 µg/ml chloramphenicol at 30 °C [14, 17]. Both the generated complement strain: the *mazE* mutant/pCL55 carrying *mazE* and the *mazF* mutant/pCL55 carrying *mazF* were used as a control for several experiments. The complement genes in the mutants were verified by performing PCR and sequencing.

### Generation of *lrgB* overexpressing *mazE* mutant strain

To construct the tetracycline-inducible *lrgB* in vector pYJ335, a DNA fragment containing *lrgB* with its ribosome binding site was amplified from the genome of *S. aureus*. The PCR amplified fragment was treated with T4 polynucleotide kinase and was ligated into EcoRV digested pYJ335 vector such that it lies downstream of the *xyl-tetO* promoter. The ligation product was transformed into *Escherichia coli* DH5α (Invitrogen) and the transformants were screened by performing colony PCR. Since EcoRV produces a blunt end, the correct orientation of the insert was verified by sequencing. The generated plasmid pYJ335 carrying *lrgB* gene was further transduced into *mazE* mutant *S. aureus* strain using the protocol described in Additional file 1.

### Growth curve analysis of the generated mutant strains

To check any growth defect of the generated mutant strains, 50 µl of each of the mutant cells along with the wildtype were inoculated in 5 ml TSB in a culture tube, incubated at 37 °C for 16 h overnight. The overnight cultures were diluted in 50 ml of fresh TSB in 100 ml of the conical flask to OD_600_ = 0.1 and incubated at 37 °C with constant shaking at 150 rpm. At every 1-h interval, 1 ml aliquots of wildtype and both the mutant cells were harvested in a plastic cuvette and the optical density OD was measured at 600 nm using a spectrophotometer (Shimadzu UV-1800). The OD_600_ of the bacterial growth culture was measured in biological replicates and is represented as mean ± S.D.

### Static biofilm formation assay

We followed the quantitative gold standard microtitre plate method for the quantification of biofilm formation in the wildtype, the two mutant strains along with the complement strain (the *mazE* mutant/pCL55 carrying *mazE* and the *mazF* mutant/pCL55 carrying *mazF*) [24]. Isolated colonies of each strain were inoculated in trypticase soy broth (TSB) with 1% glucose for 24 h at 37 °C. Then 200 μl of (1:100) diluted cultures were transferred to individual 96 well plates (Tarson, India). In order to find the role of *cidA* controlled cell lysis, DNaseI treatment was performed. 28 units of DNaseI were added to each of the wells. The 96 well-plates were then incubated statically at 37 °C for 24 h. After incubation, the wells were washed thoroughly with PBS solution. Biofilm formed by bacteria adherent to the wells was fixed and was then stained by 0.1% crystal violet. After staining for 15 min, the adherent cells in the well were dissolved in 30% acetic acid. The absorbance of dissolved, stained adherent biofilm was monitored at wavelength 570 nm, using a micro-ELISA autoreader (Model 680, Biorad, UK). For the blank, uninoculated wells containing TSB were used. The absorbance values used for reporting biofilm production were blank corrected. The percentage of cells within the biofilm was calculated by determining the correlation between the cell growth (OD_600_ nm) and crystal violet absorbance (OD_570_ nm). The experiment was performed in triplicate.

### CLSM microscopy for analyzing dead cells from the static biofilm

200 μl cultures of wildtype and the two mutants were grown at 37 °C for 24 h in 96 well plates. Then, the supernatants were thrown, and the unwashed biofilms for each were resuspended in 3% (w/v) NaCl solution. The samples for each strain were harvested into an eppendorf tube and centrifuged at 10,000 rpm for 2 min. To wash the cell pellets, 0.85% (w/v) NaCl was used, followed by resuspension in propidium iodide (PI) (4 µM). The cell density after resuspending in 0.85% (w/v) NaCl solution enabled efficient counting of single cells. A drop of each cell suspension was placed on a glass slide under a coverslip, and six field views per slide were obtained by means of confocal laser scanning microscopy (CLSM) using Leica TCS-SP8 STED (Leica Microsystems—a Division of DHR Holding India Pvt. Ltd.) super-resolution confocal microscope, under ×1000 magnification. The red fluorescence exhibited by the dead cells was detected by excitation with a 522-nm argon-krypton laser, and the emission was collected with a 580-nm to 630-nm bandpass filter. The total cell number was sensed by differential interference contrast (DIC) keeping excitation at 522 nm, and collecting emission with a 505-nm long-pass filter. Image procurement was done by using Leica Application Suite X (LAS X). After counting cells in all six fields of view, the total red cells number was divided by the total cells (red cells + unstained cells) and multiplied by 100 to calculate the dead cell percentage for each sample. For each experiment, 500–1000 cells were counted per slide as described previously using ImageJ software [25].

### Flow cytometry of static biofilm

The wildtype and mutant bacteria were grown into biofilm as described above. The cell pellets were washed with 0.85% (w/v) NaCl solution and stained with 0.1% propidium iodide (PI) in the buffer. The Flow cytometry measurements were taken on a FACS BD LSRFortessa X20 Cell Analyzer (Becton Dickinson) with the threshold set to side scatter (SSC) and flow rate set to the lowest possible, 6 µl/min. All measurements run for 10,000 events. Parameters were used as follows: excitation by a 20 mW, 488 nm laser; detection filter of 695/40 nm bandpass filter. Data were averaged over three identical experiments.The percentage of dead cells was analyzed using BD FACS-Diva™ software (Becton Dickinson).

### Antibiogram profiling

All isolates, including the complement strains, were assessed for antibiotic sensitivity using VITEK2 (bioMérieux). The sensitivity against different antibiotics like benzylpenicillin, oxacillin, gentamicin, ciprofloxacin, levofloxacin, erythromycin, clindamycin, linezolid, daptomycin, teicoplanin, tetracycline, tigecycline, rifampicin, trimethoprim/sulphamethoxazole was tested. Vancomycin, linezolid macro-E-tests, and D-tests (to verify inducible clindamycin resistance) were performed, following standard methods [26].

### Colony hemolysis assay

Overnight grown cultures of wildtype P-1780 along with the two mutants and the complement strains were diluted to OD_600_ 0.05. Then 2 µl of the cultures were spotted on the freshly prepared 5% sheep blood agar plate. The blood agar plate was incubated overnight at 37 °C.

### Quantitative blood hemolysis assay

PBS solution was used to wash the human erythrocytes until the supernatant was clear. Washed erythrocytes were suspended in PBS to create a 3% (v/v) solution. 70 µl of the freshly diluted 3% RBC solution was aliquoted into 96 well plates containing 30 µl of the overnight grown culture of different strains of wildtype, *mazE* mutant*, mazF* mutant, and the complement strain. The initial OD_600_ of the cell suspension was made to 0.9 for all the strains used in this experiment. Plates were incubated statically at room temperature for 1 h. Hemolytic activity was evaluated by measuring the OD at 540 nm using a plate reader. For positive control and negative control, 1% Triton X 100 and growth medium were used, respectively [27].

### Urease activity

Urease production was assayed on urea agar slants (Himedia, India.) according to standard protocol. In this test, urease-positive strains will show a color change from orange to pink, while urease-negative strains will show no color change. An equal amount of overnight grown three different MRSA strains (wildtype, *mazE* mutant, and the complement *mazE* mutant/pCL55 carrying *mazE* gene) was stabbed and streaked onto the agar slant and was kept overnight at 37 °C.

### Animal experiment

The wildtype, *mazE* mutant, and *mazF* mutant of the P-1780 strain were grown in TSB at 37 °C overnight. The overnight cultures were diluted 100 times with fresh TSB and further incubated until OD_600_ = 1.0. Cells were pelleted by centrifugation and washed with PBS solution. Cells were suspended in PBS, and the cell density was adjusted to OD_600_ = 2.0. The bacterial suspension (50 µl) was administered into sex-matched ten C57BL/6 mice (8-week-old) via retro-orbital route. Three days later, the mice were euthanized, and the kidneys and liver were harvested and grounded. The ground organs were diluted and spread on TSA to enumerate bacterial load.

For the long-term survival experiment, the experiment was carried out as described above, except that the bacterial cell density was adjusted to OD_600_ = 1.0. After retro-orbital administration of the bacterial suspension (50 μl) into sex-matched six C57BL/6 mice (8-week-old), the mice were watched for 14 days. During the experiment, all moribund mice were euthanized immediately.

### Total RNA extraction for RNA-sequencing and RT PCR

Total RNA was extracted from overnight grown three different strains: wildtype, *mazE* mutants*,* and *mazF* mutants using the RNeasy plus mini kit (Qiagen) following the manufacturer’s protocol with slight modification. Cell lysis was done with lysostaphin (1 µg/ml) at 37 °C/10 min followed by vigorous mixing and bead beating. After removing the genomic DNA contamination by DNaseI treatment, the RNA samples were eluted in 60 µl of DEPC treated water. The concentration of the eluted RNA was measured using the Nanodrop spectrophotometer. Out of 60 µl of total RNA, 30 µl was used for the RNA-seq experiment (transcriptome analysis) while the remaining portion was used to make cDNA for qPCR analysis.

### Bioinformatics and transcriptome analysis

The RNA sequencing data for wildtype and *mazE* mutant was outsourced from the company (AgriGenome Labs Private Limited, Kochi, Kerala). Analysis of three sets of biological replicates was carried out. The fastq files obtained from the sequencer (Illumina HiSeq4000) were pre-processed prior carrying out assembly. Adapter removal and subsequent quality trimming based on quality cut-off Q30 were done using AdapterRemoval-V2 tool [28]. Further, the rRNAs were excluded by aligning with the SILVA database utilizing bwa-aligner [29]. The cleaned reads were assembled by Trinity with default settings, which generated 3553 transcripts [30]. Then the RNA-sequencing reads were mapped with UniProt database using BLASTX program with E-value cutoff of 10^–3^, against the reference database of *S. aureus* USA300_FPR3757. Transcript quantification of individual samples is carried out with the Salmon tool by using the corresponding estimation perl script available with trinity [31]. Genes differentially expressed among these two samples: wildtype and *mazE* mutants were identified using the numbers of mapped reads as EdgeR inputs [32]. Statistical analysis was performed using DESeq2, where genes with an adjusted p-value ≤ 0.05, FDR ≤ 0.001, and fold change ≥ 2 were identified as being differentially transcribed [33].

### RT-PCR validation

Reverse transcription was performed with the random hexamers using 10 µg of the eluted RNAs from the three samples. The total RNA was checked for genomic DNA contamination before performing a reverse transcription reaction by performing PCR amplification with it. The absence of any bands post PCR amplification indicated the absence of any gDNA contamination. The qPCR primers were designed using Primer3 software and tested for efficacy with genomic DNA as a template. Quantitative PCR analysis was carried out with the iTaq Universal SYBR Green Supermix (Biorad) using the CFx96 Touch Real-time PCR machine (Biorad). The following program was performed: 30 s at 95 °C, 40 cycles of 10 s at 95 °C and 30 s at 50 °C/55 °C depending upon the primers used. The Cq (quantification cycle) values were automatically determined by the CFX Maestro software after 40 cycles followed by melting curve analysis. The Cq values obtained for each of the genes were normalized against the housekeeping gene *gyrB* of *S. aureus.* For each of the genes, the analysis was carried out for at least three biological replicates.

### Statistical analysis

The statistical analysis was carried out with GraphPad-Prism software version 5.01. For continuous variables unpaired Student’s *t*-test and for categorical variables Mann–Whitney test was done. The results were considered statistically significant if the p-value; *p* ≤ 0.05 (two-tailed).

## Results

### Construction of *mazE* and *mazF* mutants by pnCasSA-BEC system

**Fig. 1:**
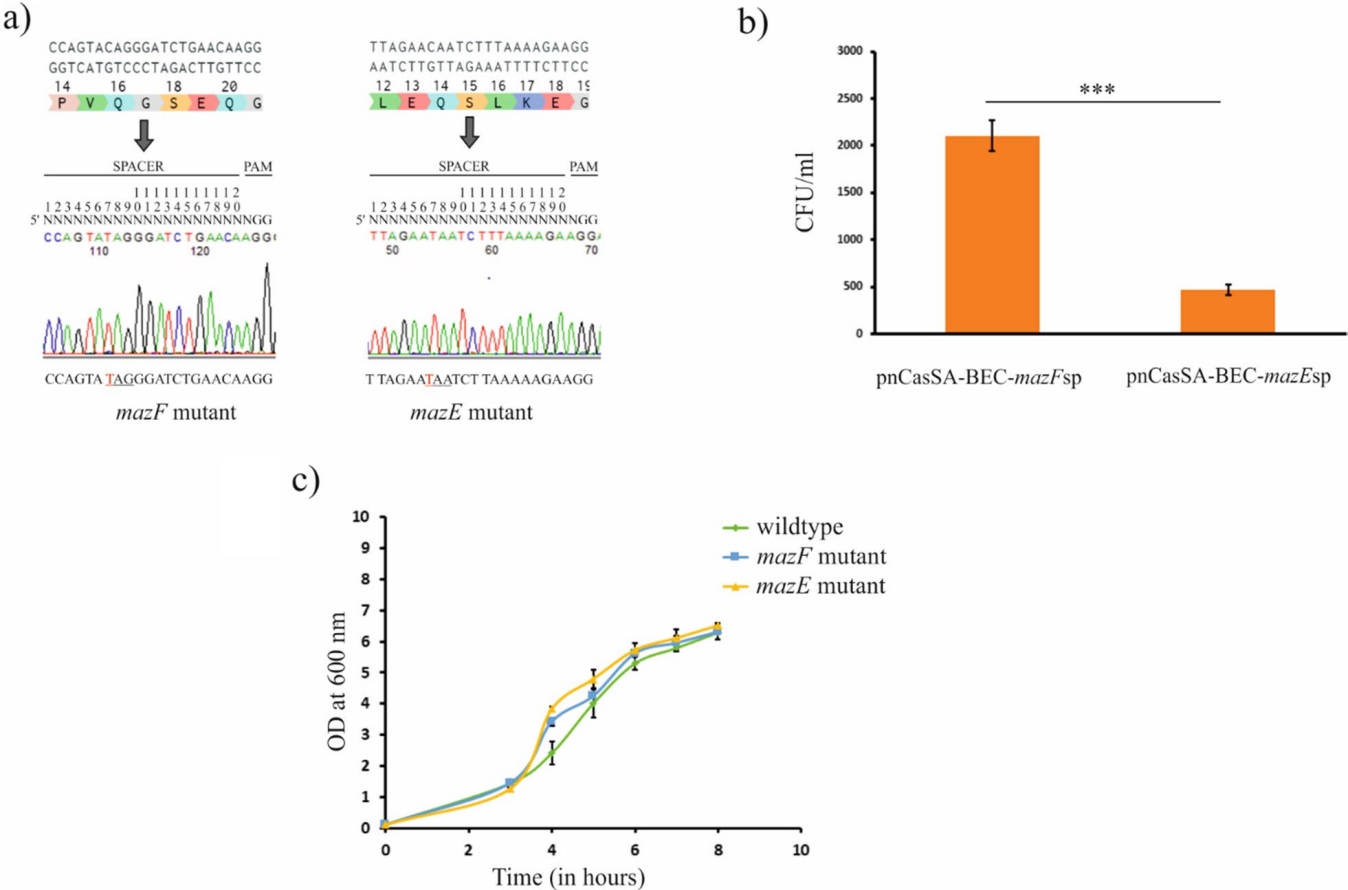
Mutation of *maz*EF TA gene and its effect on growth rate in MRSA P-1780 strain. **a** Sequencing chromatogram revealed successful point mutation of C to T (marked in red) converting Q14 of *mazE* and Q16 of *mazF*, into a premature stop codon respectively. **b** Reduction in CFU count/ml post 16 h growth, after the transduction of the pnCasSA-BEC-*mazE*sp plasmid generating *mazE* mutants compared to *mazF* mutants. **c** Growth curve analysis of the wildtype, *mazE* and *mazF* mutants grown at 37 °C for 8 h in the TSB broth, for analyzing any growth defects

The pnCasSA-BEC plasmid system was used in the conversion of nucleotide base ‘C’ to ‘T’ in ST 239 (P-1780) clinical MRSA strain to generate a premature stop codon. The spacers of *mazE* and *mazF* genes that contain editable ‘C’ at position ‘7’ were assembled (Fig. 1a). The editable ‘C’ was converted to nucleotide ‘T’ for generating a premature stop codon. Since the transformation efficiency in ST239 MRSA strains is very low, assembled plasmids were transduced to wildtype P-1780 with the help of phage phi85. The CFU/ml count was significantly reduced (t-test, p-value < 0.001) for transduced *mazE* mutant cells compared to *mazF* mutant as shown in Fig. 1b. Thus, the active MazF toxin confers programmed cell death (PCD) on the P-1780 cells. The Q 16 of *mazE* and Q 14 of *mazF*, were mutated to stop codon successfully, as shown in Fig. 1a. The pnCasSA-BEC possesses the temperature-sensitive origin of replication (*repF*). Therefore, the same method was used to cure the plasmid as mentioned previously [14]. After growing the cells at a non-permissive temperature (42 °C), all the arbitrarily chosen colonies were seen to grow in the absence of chloramphenicol but were unable to grow with its presence, suggesting the effective removal of the pnCasSA-BEC from the cells.

### Growth characteristics of *mazE* and *mazF* mutants

The wildtype, *mazE* and *mazF* mutant strains were further tested for their growth characteristics by generating bacterial growth curves at various time intervals. The growth curve analysis (Fig. 1c) shows no change in the growth pattern in the case of both the mutants when compared to the wildtype strain, suggesting that mutants do not have any growth defects.

### Differentially expressed genes from transcriptomics analysis

**Fig. 2:**
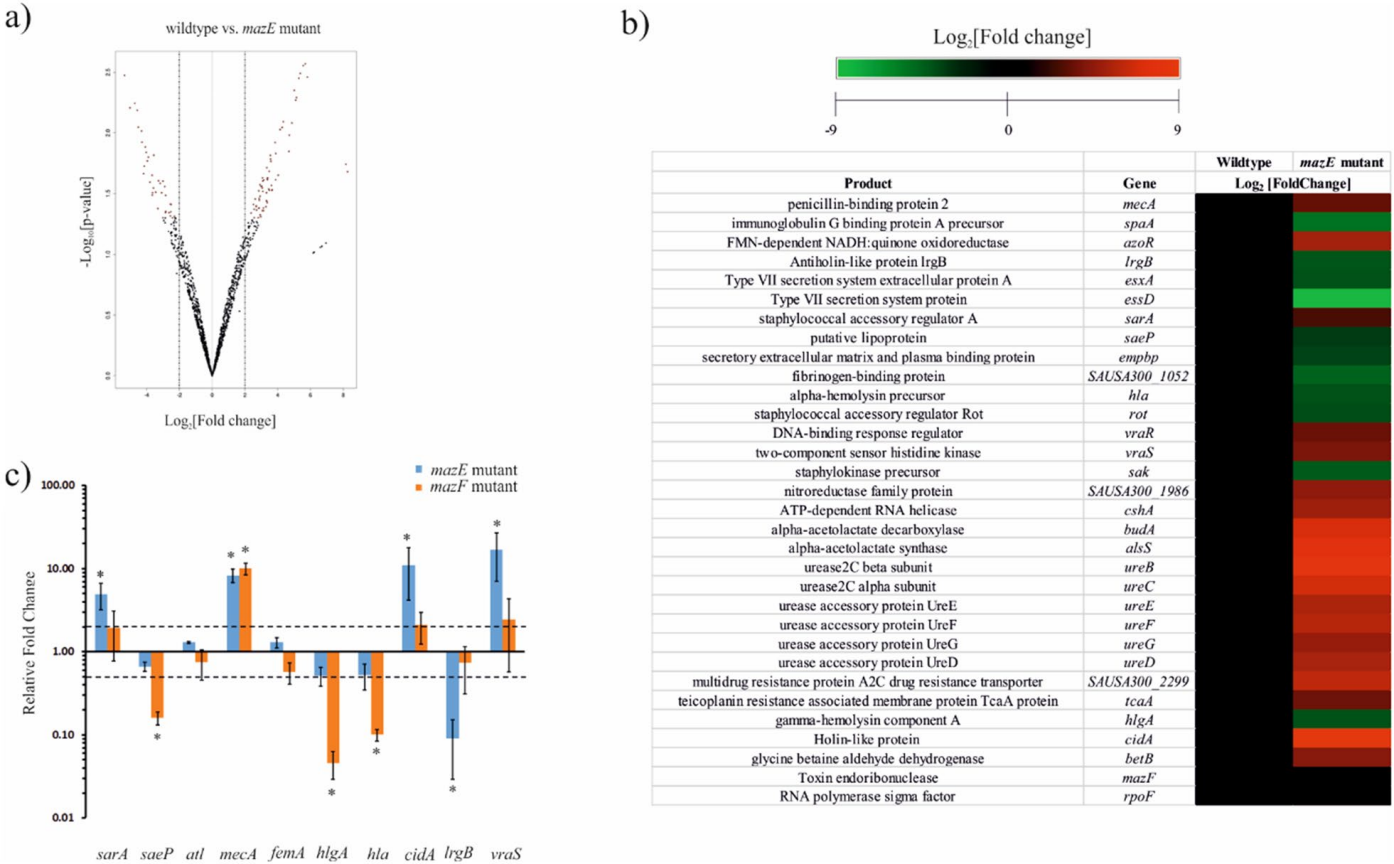
Differentially expressed genes in the P-1780 MRSA *mazE* mutant strain. **a** Volcano plot showing the differentially expressed genes in case of wildtype vs *mazE* mutant, after transcriptome analysis. **b** Heat map representation of some of the important genes, showing significant (p-value < = 0.05) differential expression. **c** qPCR analysis to check the differentially expressed genes in P-1780. Relative gene expression of different genes after *mazE* and *mazF* gene mutation in the MRSA strain. Error bars represent the standard deviation. Stars (*) above the error bars indicate the genes which are significantly differentially expressed (0.5 ≥ Fold change ≥2)

From the RNA-sequencing data, we found that a total of 178 genes were differentially regulated in the case of wildtype vs. *mazE* mutant strain, which was statistically significant. Out of the 178 genes, 65 genes were found to be upregulated, while 113 genes were downregulated. The differentially expressed genes are indicated in the volcano plot (Fig. 2a). This observation indicates that mutation of *mazE* generates an alteration in the pattern of gene expression profile in the clinical MRSA strain (Additional file 1: Table S2). Some of the genes affected by the *mazE* mutation are shown as heat map representation (Fig. 2b). Mutation in *mazE* gene lead to significant upregulation of the holin encoding *cidA* gene responsible for cell lysis induced biofilm formation with a concomitant reduction in the expression of *lrgB* gene encoding the anti-holin protein. The CidA protein forms a pore in the membrane through which CidB autolysin can easily pass, thus rupturing it and causing PCD [34]. On the other hand, the LrgB protein works in a contrary manner whereby it blocks the murein hydrolase activity of CidA [34]. Interestingly, in the *mazE* mutant, cell-lysis induced by *atl* gene encoding the autolysin protein was found to be downregulated. These autolysins are responsible for *ica* and PIA independent biofilm formation in clinical MRSA strain by its catalytic activity of the amidase region [35, 36]. On the other hand, *sarA*, another biofilm regulatory gene, known to contribute to persistent endovascular infection by promoting biofilm formation in MRSA cells showed upregulation in the *mazE* mutant strain [37, 38]. Also, the increased expression of the *sarA* and *cshA* gene, along with the *cidA* gene, in the *mazE* mutant strain remarkably points towards a tendency to form biofilm [37, 39] The *alsS* and the *budA* gene, responsible for mitigating nitrosative stress in *S. aureus*, were also seen to be upregulated in the generated mutants signifying that *mazF* helps in managing nitrosative stress [40]. Similarly, the *betB* gene was upregulated in the *mazE* mutants, which acts as a compatible solute in response to osmotic shock [41]. The urease BCDEFG operon, responsible for maintaining intracellular pH homeostasis by converting urea to ammonia and carbon dioxide, was found to be highly upregulated in the *mazE* mutants, showing that *mazF* promotes tolerance to acid stress, which arises from formate metabolism [42]. The *rot* (repressor of toxins) acts as a global regulator of virulence genes was downregulated [43]. Genes like *mecA*, *vraS*, *vraR, tcaA* and multidrug resistance proteins responsible for promoting antibiotic resistance were also significantly upregulated in the *mazE* mutant strains. On the other side, genes responsible for host–pathogen interaction and virulence like *hlgA*, *empbp*, *hla*, *essD*, *esxA*, *rot*, *sbi*, *saeP*, *saeR*, *sak*, *spa,* and fibrinogen binding protein showed significant downregulation in the mutants studied. The *azoR* gene responsible for providing resistance to thiol-specific stress was upregulated in *mazE* mutants, suggesting the role of *mazF* in the thiol stress response system [44]. Thus, a mutation in the *mazE* gene caused a change in the expression patterns of a plethora of genes that take part in various molecular and cellular pathways in the clinically isolated ST239 MRSA strain.

### Differentially expressed genes from qPCR analysis

It has already been reported that *mazF* selectively cleaves mRNAs in vivo, avoiding essential transcripts like *recA*, *gyrB* and *sarA* in *mazF* induced cells [45]. So, we performed the qPCR analysis of the *sarA*, *saeP*, *atl*, *hlgA, hla, cidA, lrgB mecA*, *femA* and *vraS* gene using *gyrB* as the reference gene. From the qPCR analysis (Fig. 2c), the *sarA* gene was found to be 4.88-fold upregulated in the *mazE* mutant, compared to wildtype strain. No significant change in *sarA* expression was observed in the *mazF* mutant strain. The *saeP* gene was found to be downregulated in both the *mazE* and *mazF* mutant strains. The change in the *atl* (autolysin) gene in both the mutant strain was not significant. The *mecA* gene responsible for cefoxitin/oxacillin resistance was upregulated around eightfold and tenfold in the case of *mazE* and *mazF* mutants respectively. For the *fem* gene, no significant change was observed for both the mutant strains. The *hlgA* and the *hla* gene were found to be downregulated in the case of both the *mazE* and *mazF* mutants. The *cidA* and *vraS* genes were seen to be upregulated 10.9-fold and 12.3-fold respectively in the *mazE* mutant strain whereas no significant change in expression was observed for the *mazF* mutant strain. Also, *mazE* mutant revealed significant downregulation of the *lrgB* gene with no expression change in case of *mazF* mutant. The validity of the qPCR data was confirmed by melting curve analysis of the above mentioned genes (Additional file 1: Fig. S3). Thus, the qPCR analysis clearly highlights the change in gene expression caused by mutation of the *mazE* and *mazF* gene.

### The *mazF* induced stressful condition deregulates *cidA*, leading to cell lysis promoting biofilm formation in MRSA P-1780

**Fig. 3:**
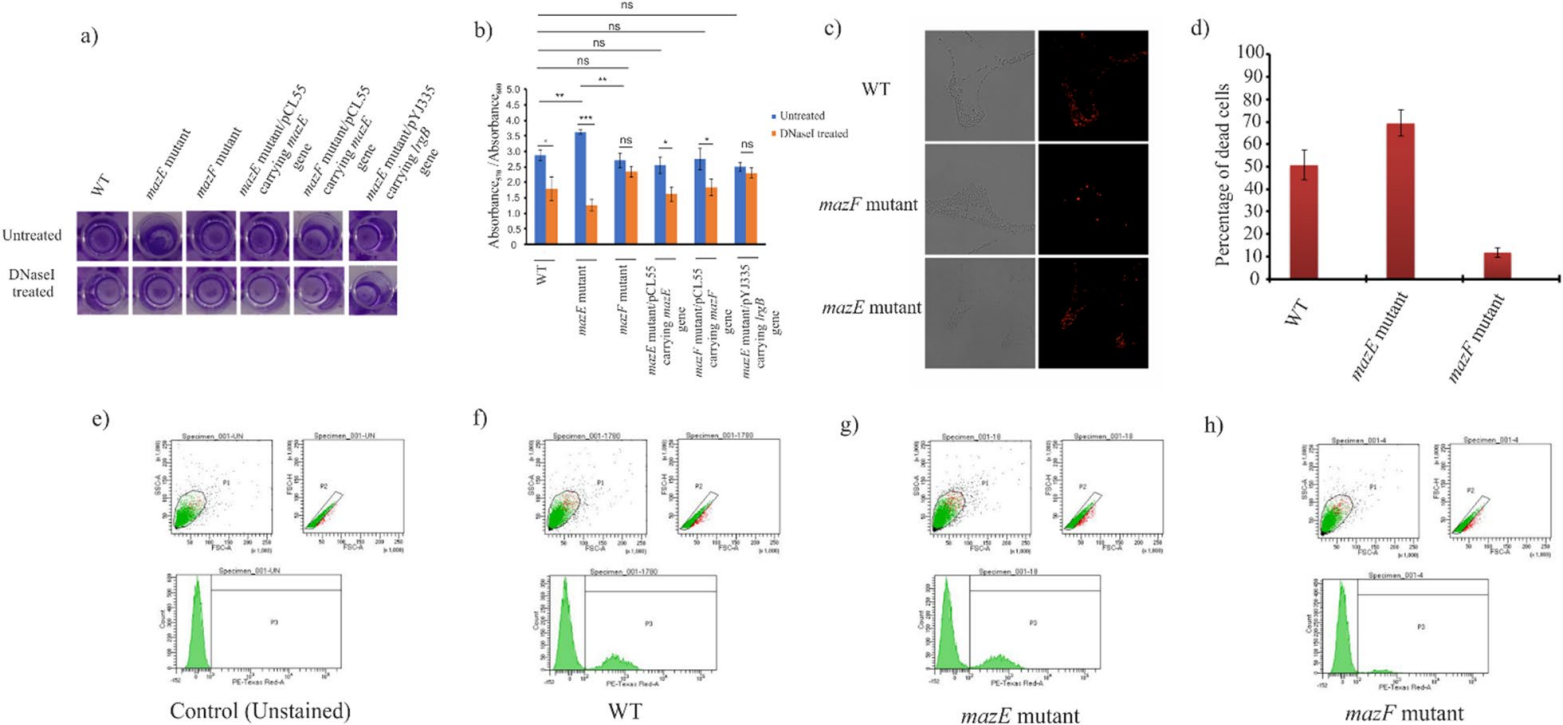
Cell death-induced biofilm formation by the different test strains. **a** Microtitre-well showing biofilm formation in the wildtype along with both the mutants, complement strains and the *lrgB* overexpressing *mazE* mutant after crystal violet staining: with untreated and DNaseI treatment. **b** The *mazE* antitoxin mutant showed increased biofilm formation in case of untreated while, DNaseI treatment drastically reduced the biofilm formation in case of wildtype and *mazE* mutant strain. The graph depicts the correlation of the measured crystal violet absorbance of the attached cell (Absorbance 570 nm) to the planktonic cell growth (Absorbance 600 nm). Each point and the standard deviation are the measures of three independent samples per condition. (t-test: ‘*’ denotes p-value between 0.01–0.05: significant; ‘**’ denotes p-value between 0.001–0.01: highly significant; ‘n.s’ denotes not significant). **c** Confocal laser scanning microscopy of the three test strains obtained from the biofilm, after staining with 4 µM propidium iodide (PI). Dead cells appear as discrete red puncta when excited with a 522-nm argon-krypton laser and emission collected with a 580-nm to 630-nm bandpass filter. **d** Percentage of dead/total biomass were calculated for the MRSA cells from the biofilm sample. Each point and the standard deviation is the measure of three independent samples per condition. Flow cytometry analysis for **e** Control (unstained), **f** wildtype, **g** *mazE* mutant, **h** *mazF* mutant, were done with the following settings: threshold set to side scatter (SSC) and flow rate set to the lowest possible, 6 µl/min. All measurements were run for 10,000 events. Data were averaged over three identical experiments. The percentage of dead cells was analyzed using BD *FACS Diva*™ (Becton Dickinson)

Biofilm formation assay revealed that both the wildtype and the generated mutants were successfully able to form biofilm. However, as shown in Fig. 3a and b, the biofilm formation was increased in the *mazE* mutant strain (Absorbance_570/600_ 3.62, p-value < 0.01, t-test). On the other hand, the *mazF* mutant showed decreased biofilm formation (Absorbance_570/600_ 2.71, p-value > 0.05, t-test). The biofilm formation in the *mazE* complement (*mazE* mutant/pCL55 carrying *mazE* gene) and the *mazF* complement strain (*mazF* mutant/pCL55 carrying *mazF* gene) were found to be similar to the wildtype (Absorbance_570/600_ 2.88). These results shows that *mazF* toxin enhances biofilm formation in the P-1780 strain. To evaluate the importance of *cidA* mediated cell lysis for the release of eDNA, the biofilms of wildtype, *mazE* and *mazF* mutants along with the complement strains were treated with DNaseI at the time of inoculation and was further grown for 24 h. DNaseI treatment tremendously decreased *mazE* mutant biofilm compared to untreated biofilm (p-value < 0.001, Students t-test) (Fig. 3b). This observation pointed to the fact that a higher percentage of the cells involved in the biofilm formation in *mazE* mutant was dead and lysed, which might be due to the activated MazF*,* which leads to upregulated *cidA* and downregulated *lrgB* (as obtained from the transcriptomics data). To ascertain the fact that the increased biofilm formation in *mazE* mutant is due to upregulated *cidA*, we tried to overexpress *lrgB* gene (inhibitor of *cidA*) in the *mazE* mutant background and checked the biofilm formation [34]. The biofilm formation was significantly reduced (Absorbance_570/600_ 2.5) in the *lrgB* overexpressing *mazE* mutant strain compared to only *mazE* mutant (Fig. 3b). However, DNaseI treatment did not affect the biofilm formation in the *lrgB* overexpressing *mazE* mutant strain. To further examine whether the biofilm formation by the *mazE* and *mazF* mutants correlates with cell death, we measured the dead cells in the biofilm by propidium iodide (PI) staining (Fig. 3c). In the wildtype MRSA strain, the percentage of dead cells was found to be 50.7%. However, in the case of the mutant strains, the percentage of dead cells was 69.4% (*mazE* mutant) and 11.6% (*mazF* mutant), respectively (Fig. 3d). FACS analysis also showed a similar pattern where the percentage of dead cells is higher in *mazE* mutant strain (26.2%) when compared to *mazF* mutant (7.9%) (Fig. 3e–h). Thus it can be hypothesized that *mazF* toxin works synergistically with *cidA* in regulating cell death and lysis mediated biofilm formation.

### MazF toxin changes MRSA P-1780 cell susceptibility towards β-lactams, glycopeptide vancomycin, and the lipopeptide daptomycin

From the VITEK2 antibiogram profiling, the susceptibility pattern of the five different test strains was checked in presence of different antibiotics. For the wildtype and the complement strains (the *mazE* mutant/pCL55 carrying *mazE* and the *mazF* mutant/pCL55 carrying *mazF*), the MICs for oxacillin, daptomycin, and vancomycin observed were 1 µg/ml, < = 0.5 µg/ml and < = 0.5 µg/ml respectively, but for *mazE* mutant, the MIC for oxacillin, daptomycin and vancomycin was increased to > = 4 µg/ml, > = 8 µg/ml, > = 32 µg/ml respectively, confirming the reduction in oxacillin, daptomycin and vancomycin susceptibility. But rest of the antibiotics including gentamicin, ciprofloxacin, levofloxacin, erythromycin, clindamycin, linezolid, teicoplanin, tetracycline, tigecycline, rifampicin, trimethoprim/sulphamethoxazole showed no significant changes in MIC values (Table 1). Similar results supporting antibiotic resistance were obtained from transcriptome analysis (RNA-seq) which showed significant downregulation in transcription of cell surface protein genes (*spa*), second immunoglobulin-binding protein (*sbi*), regulatory genes (*saePRS*), PP2C phosphatase gene with upregulation in multi-drug resistance protein, betaine aldehyde dehydrogenase (responsible for the accumulation of the compatible solute glycine, betaine) and *ure* genes of the urease operon. RNA-sequencing results showed characteristic expression profiles for *vraS* and *vraR,* which were up-regulated in the *mazE* mutants, confirming the changed phenotype of glycopeptide antibiotic resistance [46]. The penicillin-binding protein 2A (*mecA*), responsible for methicillin resistance was upregulated in the *mazE* mutant, suggesting a possible role of the *mazF* toxin in regulating resistance to beta-lactams or cephalosporin group of antibiotics. Thus, it can also be demonstrated that the *mazF* toxin is responsible for making the P-1780 strain resistant to beta-lactam, glycopeptides and lipopeptide group of antibiotics.

**Table 1:** Table showing different antibiotic response of the WT P1780 strain and the generated *mazE* mutant, *mazF* mutant and the complement strains: *mazE* mutant/pCL55 carrying *mazE* gene and *mazF* mutant/pCL55 carrying *mazF* gene

	Wildtype		*mazE* mutant		*mazF* mutant		*mazE* mutant/ pCL55 carrying *mazE* gene		*mazF* mutant/ pCL55 carrying *mazF* gene	
Anti-microbials	MIC	Interpretation	MIC	Interpretation	MIC	Interpretation	MIC	Interpretation	MIC	Interpretation
Benzylpencillin	> = 0.5	R	> = 0.5	R	> = 0.5	R	> = 0.5	R	> = 0.5	R
Oxacillin	1	R	> = 4	R	> = 4	R	1	R	1	R
Gentamicin	> = 16	R	> = 16	R	> = 16	R	> = 16	R	> = 16	R
Ciprofloxacin	> = 8	R	> = 8	R	> = 8	R	> = 8	R	> = 8	R
Levofloxacin	4	R	4	R	4	R	4	R	4	R
Erythromycin	> = 8	R	> = 8	R	> = 8	R	> = 8	R	> = 8	R
Clindamycin	> = 4	R	> = 4	R	> = 4	R	> = 4	R	> = 4	R
Linezolid	2	S	2	S	2	S	2	S	2	S
Daptomycin	< = 0.5	S	> = 8	R	1	S	< = 0.5	S	< = 0.5	S
Teicoplanin	< = 0.5	S	< = 0.5	S	< = 0.5	S	< = 0.5	S	< = 0.5	S
Vancomycin	< = 0.5	S	> = 32	R	1	S	< = 0.5	S	< = 0.5	S
Tetracycline	> = 16	R	> = 16	R	> = 16	R	> = 16	R	> = 16	R
Tigecycline	< = 0.12	S	< = 0.12	S	< = 0.12	S	< = 0.12	S	< = 0.12	S
Rifampicin	< = 0.03	S	< = 0.03	S	< = 0.03	S	< = 0.03	S	< = 0.03	S
Trimethoprim/sulfamethoxazole	> = 320	R	> = 320	R	> = 320	R	> = 320	R	> = 320	R

*R* Resistant, *S* Sensitive

### *mazEF* system affect the hemolysis activity of P1780

**Fig. 4:**
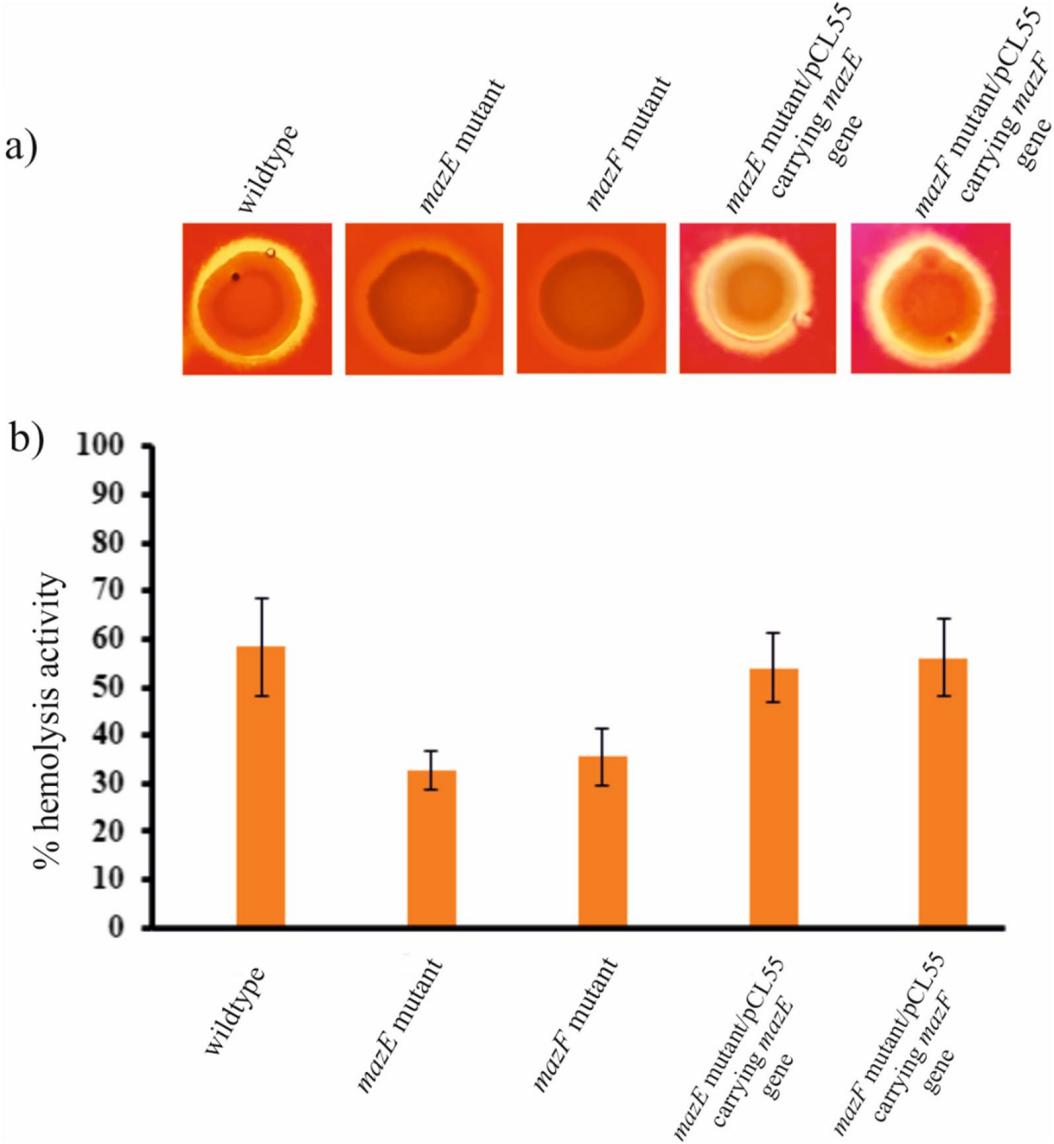
Beta hemolytic activity shown by the P-1780 MRSA strains. **a** Liquid cultures of wildtype, *mazE* mutant*,* and *mazF* mutant along with both the complement strains were spotted onto blood agar plates containing 5% sheep blood and incubated overnight at 37 °C. **b** Percentage hemolysis of red blood cells were calculated for the five test strains by measuring the absorbance at 540 nm. Error bars represent standard deviation. Each point and standard deviation are the measures of three independent samples per condition

Spot plate hemolysis assay on blood agar plates revealed that the wildtype, *mazE* and *mazF* mutants, as well as both the complement strains, were capable of lysing red blood cells (discoloration of red color surrounding the colonies) (Fig. 4a). However, the beta-hemolysis shown by the *mazE* mutant strain and the *mazF* mutant strain was found to be less than that of the wildtype. Moreover, both the complement strain showed a similar hemolysis pattern as the wildtype. Since the spot plate assay was qualitative, we performed a quantitative blood hemolysis assay using a 3% human RBC solution. We observed different percentages of hemolytic activity for all the tested MRSA strains. The percentage hemolysis calculated for the wildtype, *mazE* mutant and *mazF* mutant strains was 58.32%, 32.76% and 35.51%, respectively (Fig. 4b). While the percentage hemolysis for the *mazE* mutant/pCL55 carrying *mazE* and the *mazF* mutant/pCL55 carrying *mazF* were observed as 54.02% and 56.23% respectively. From the graph, it is evident that there was a significant change in the percent hemolysis of the *mazE* and *mazF* mutants when compared to the wildtype. This observation from both experiments suggests that the *mazEF* toxin–antitoxin system affects the hemolysis activity of the MRSA P-1780 strain. This was also confirmed by the decreased expression of *hla and hlgA* gene responsible for hemolysis (Fig. 6, Additional file 1: Table S2) [38].

### Urease operon is positively upregulated by *mazF* toxin

**Fig. 5:**
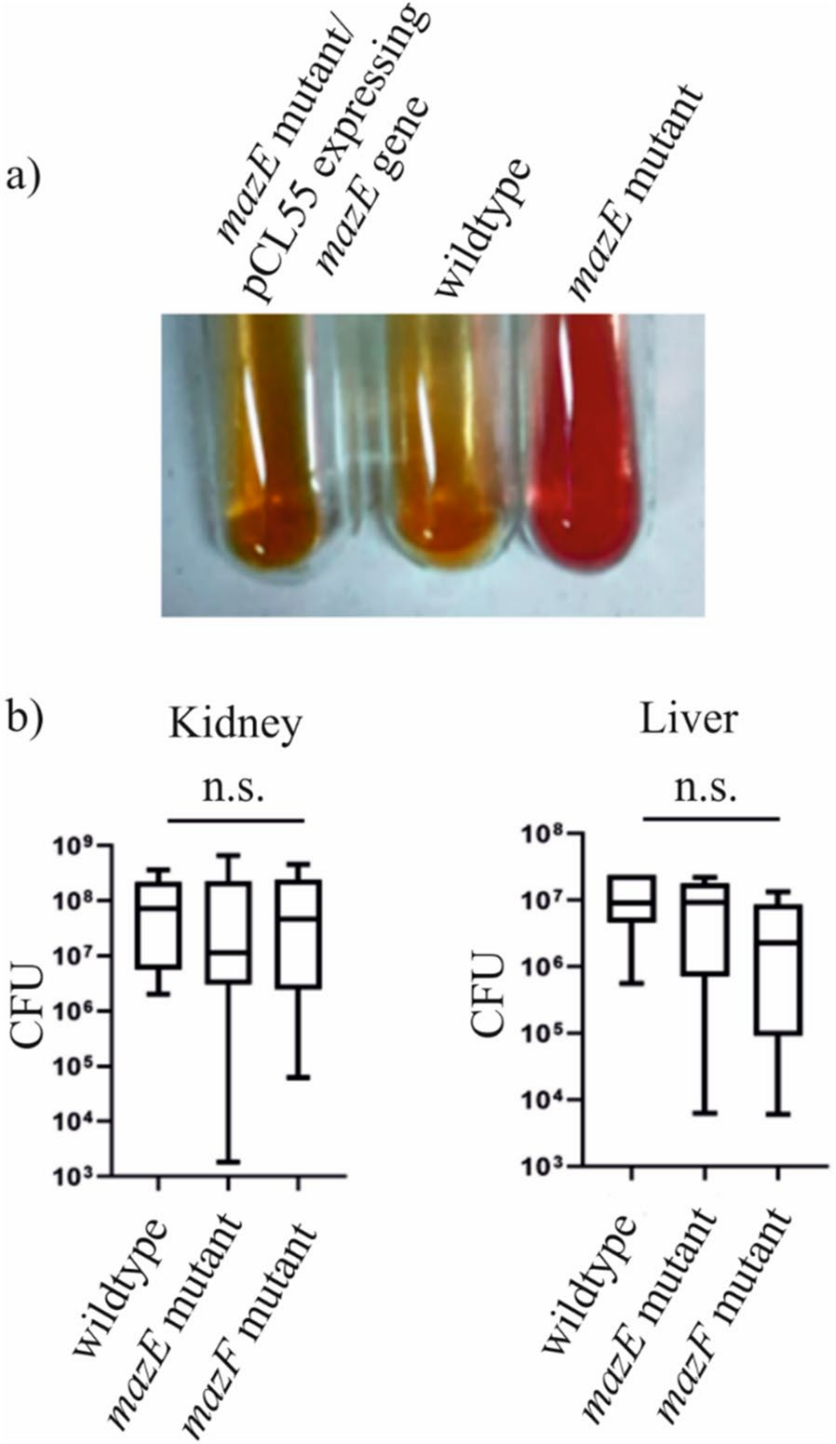
Virulence activity shown by the P-1780 MRSA strains. **a** Overnight grown cultures of wildtype, *mazE* mutant and the *mazE* complement strains were streaked onto urea agar slant. The urease activity was monitored by the color change of the urea agar slant after 24 h of growth. **b**The wildtype, *mazE*, and *mazF* mutant were administered into ten C57BL/6 mice via retro-orbital injection. Three days post-infection, the mice were euthanized, the kidneys and livers were harvested, and CFU in the organs was counted. The statistical significance was determined by the Mann–Whitney test. n.s.: not significant

From RNA-sequence analysis, it was seen that, unlike rot, the *mazF* toxin positively regulates various urease genes like *ureB*, and *ureC*, that encode different subunits of the urease enzyme, along with the accessory genes like *ureD* and *ureE*, essential for its enzymatic activity [43]. Therefore, we also tested the urease activity on urea agar slants for the wildtype, *mazE* mutant, and *mazE* mutant/pCL55 carrying *mazE* strains. As shown in Fig. 5a, the *mazE* mutant strain possessing the activated MazF toxin was urease positive within 24 h but the wildtype and the complement strain (*mazE* mutant/pCL55 carrying *mazE*) were urease negative when kept for 24 h. The *mazF* mutant was also found to be urease negative (data not shown).

### The *mazEF* TA system is not involved in host–pathogen interaction mediated virulence

Earlier studies have identified EssD, a novel secreted effector involved in the ESS pathway, which functions as a DNA nuclease, whereby it stimulates interleukin (IL-12) signaling to confer pathogenicity in the *S. aureus*, within deep-seated abscess lesions [47]. On the other hand, in the ESAT-6-like system, *S. aureus* mutants that failed to secrete EsxA and EsxB exhibited pathogenesis defects in murine abscesses, signifying that this specialized secretion system might play an important role in human bacterial pathogenesis [48]. Both the *essD* and the *esxA* genes were found to be downregulated significantly in the *mazE* mutant strain. Since the *mazE* and *mazF* mutations affect the transcription of multiple genes (*hlgA*, *empbp*, *hla*, *essD*, *esxA*, *rot*, *sbi*, *saeP*, *saeR*, *sak*, *spa,* etc.) involved in staphylococcal pathogenesis, we examined whether those gene expression alterations, affect the virulence of *S. aureus*. The wildtype, *mazE*, and *mazF* mutant cells (~ 2 × 10^7^ cfu) were injected into ten mice via retro-orbital route. Three days later, the mice were sacrificed, and bacterial loads in the kidneys and liver were measured. As shown in Fig. 5b no significant change was observed. The *mazE* and *mazF* mutants showed altered biofilm formation (Fig. 3a and b). To examine whether the alteration of biofilm formation affects the bacterial virulence in persistence phase, we administered the reduced number of the bacterial cells (~ 1 × 10^7^ cfu) via a retro-orbital route and observed the infected mice for 14 days. As shown in Additional file 1: Fig. S4, all infected mice showed similar survival, suggesting that the gene expression alterations caused by *mazEF* mutations do not significantly affect the overall virulence of *S. aureus*.

## Discussion

**Fig. 6:**
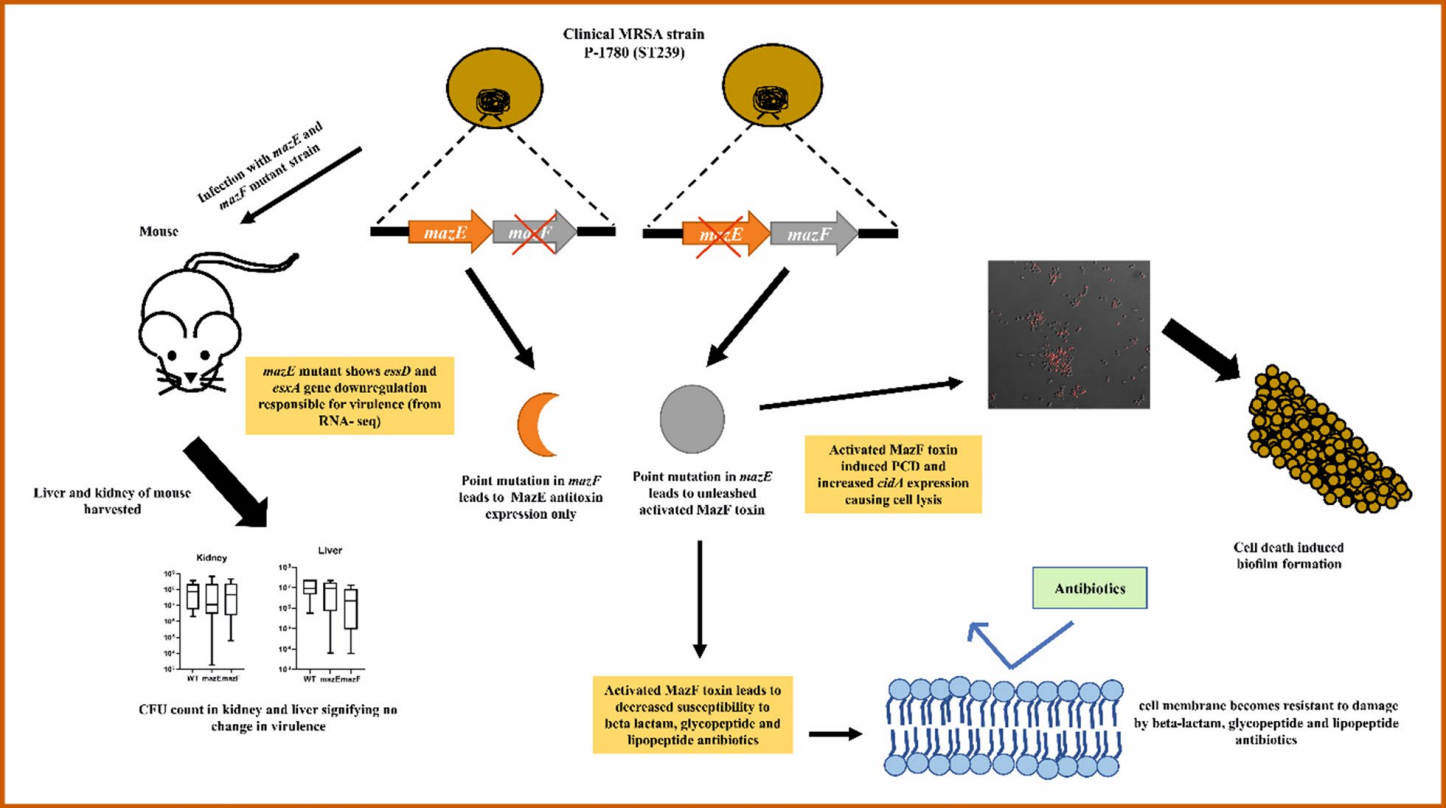
Model diagram hypothesizing the possible role of *mazEF* toxin–antitoxin system in biofilm formation, antibiotic resistance, and virulence in clinical MRSA strain

In this study, we have isolated a clinical strain of MRSA P-1780 (ST239) from pus sample of a patient and generated *mazE* antitoxin and *mazF* toxin gene mutation using CRISPR–cas9 base editing plasmid (pnCasSA-BEC) [13, 17]. The idea was to insert a premature stop codon in between the gene so that its expression gets hampered [17]. Although the *mazE* mutant strain showed a rapid decrease in CFU count when grown on plate after transduction, growth curve analysis of both the generated mutant strains confirmed their normal growth pattern. This can be possibly explained by the fact that some of the *mazE* mutant cells might have managed to survive the ectopic expression of MazF through expressing RNA binding proteins in-vivo which protects the essential and regulatory mRNA transcripts, preventing the lethal effect of MazF toxin [45]. From the transcriptomics data, the observed upregulation of RNA-binding protein genes like *cshA* and *sarA* in the *mazE* mutant might be involved in protecting the *mazF* activated cells [49, 50]. Similar results were also reported earlier by other researchers, showing the selectivity of MazF in targeting mRNAs and also pointing out that the expression of toxin is not instantly bactericidal [45]. This bactericidal effect can be reversed by the expression of antitoxin within a defined time window, otherwise, it leads to PCD [45]. We also checked the biofilm formation ability of both the mutants, where Our study reveals that activated MazF toxin leads to resistance to antibiotics like oxacillin, daptomycin and vancomycin (Fig. 6). Therefore, in the future, any potential antibacterial drug can be designed to target MazF toxin against the problematic multi-drug resistant bug. A similar study has been reported, that supports our current observation [51]. However, one of the recent studies in *S*. *aureus* (USA300 JE2) strains lacking the *mazF* gene had revealed increased biofilm growth, with decreased biofilm-associated tolerance to antibiotics [7]. Numerous *S. aureus* genes have been reported, that control biofilm-forming ability, like *cid*, *lrg*, *sarA*, *agr*, *rbf*, *sigB*, *ica*, *tcaR*, *arlRS*, and *alsSD* [25, 37, 38, 52]. The *S. aureus cidABC* and *lrgAB* operons modulate cell lysis and antibiotic tolerance oppositely: *lrgAB* plays a negative role in murein hydrolase function and enhances antibiotic tolerance, whereas *cidA* plays a positive role in murein hydrolase function and reduces antibiotic tolerance [25]. The *cidAB* and the *lrgAB* operon encode the holin-like and antiholin-like protein respectively, acting as a molecular switch to determine bacterial cell lysis [34]. From our study, the *mazE* gene mutation aggravated the expression of the *cidA* gene, inducing cell death tendency, to release the extracellular DNA necessary for biofilm formation in the mutant strain. The biofilm formation in the *mazE* mutant strain was drastically reduced in the presence of DNaseI confirming the vital role of released extracellular DNA in augmenting biofilm formation. Previous experiments performed in *E. coli* cells have shown that the presence of DNase did not reduce biofilm formation in *E. coli* suggesting that autolysin mediated cell lysis and eDNA release is not always required for bacterial biofilm formation [51]. Thus, we can speculate that the *mazF* is promoting biofilm formation via cell death, which is independent of *atl* but dependent on *cidA* holin (encoding autolysin-like protein). The *lrgAB* gene product was downregulated in the *mazE* mutant which is known to inhibit the action of CidAB protein. Thus, the increased biofilm formation in the *mazE* mutant strain can be attributed to the coordinated expression of the *cidA* and *lrgB* gene, as observed from the transcriptomics data, validated by qPCR. On the other hand, in the *mazF* mutant strain, the reduced number of dead cells justifies the decreased biofilm formation. The significance of this cell death can be attributed to the cell’s altruistic behavior, where dead cells provide nourishment to other members of the biofilm community, by releasing their cellular contents to the surrounding cells [51, 53, 54]. Thus, cells inside the biofilm can thrive for a longer period, during stress conditions. From this observation, we can speculate that the *mazF* induced *cidA* gene expression is necessary for cell death-mediated biofilm formation. Although, further studies need to be done to understand the mechanistic details of the process. The *mazEF* system is known to control the expression of its downstream *rsbUVW-sigB* operon, which encodes the sigma factor B and its regulators [55]. Also, the binding of the transcriptional regulator SarA positively activates P*mazE,* which in turn might play a role in biofilm formation [55]. By redirecting the appropriate transcription of genes, the *mazEF* system can respond to different environmental stress. It was reported earlier that increased expression of *cidBC* was dependent on the activated sigma factor B (*sigB*), while *lrgAB* expression is negatively regulated by the sigB [56]. From this, we can hypothesize that the *mazEF* system might modulate the expression of *cid* and *lrg* operon in the isolated clinical P-1780. Previously, it was reported in *S. aureus* that, inactivation of the *cshA* DEAD-box RNA helicase gene reduced biofilm formation and increased hemolysis by controlling *agr* mRNA stability [39]. The differential upregulation of the *cshA* DEAD-box RNA helicase gene in the *mazE* mutants, from the transcriptomics data, corroborated with the increased biofilm formation in the *mazE* mutant strain in comparison to wildtype with the concomitant reduced hemolytic activity of *mazE* mutated P-1780 strain (as confirmed by beta-hemolytic assay). The host–pathogen interaction and the virulence ability of the generated mutants were analyzed with the murine experiments. No significant change in the CFU counts, in the harvested liver and kidney fractions were observed in the mouse, infected individually with both the *mazE* and *mazF* mutant strain (Fig. 6). However, it was reported previously that the absence of the *mazF* toxin gene developed an increased bacterial load with reduced survival rate in mice in comparison to wildtype, indicating that the absence of the *mazF* gene triggered a rise in *S. aureus* virulence [7]. Till now, it is not known how the SaeRS two-component system (TCS) is regulated. However, the current data suggest that *mazF* is required for the upregulation of the SaeRS TCS activity with decreased hemolysis in the mutants (confirmed by blood agar assay). Further work needs to be done, which is outside the scope of this manuscript. Also, the downregulation of *rot* justifies the increased expression of the *ure* genes since rot negatively affects the urease genes [43]. Interestingly, from the antibiogram profiling experiment, we found that *mazE* mutant strain showed reduced susceptibility to oxacillin, vancomycin and daptomycin compared to wildtype. It has been documented previously that, the MRSA strains had increased expression of the *mazEF* TA gene correlating with their antibiotic resistance phenotype [19, 55]. It was also reported earlier that *mazEF* gene transcription increased when cells were exposed to penicillin, linezolid, tetracycline, and erythromycin with no change in the case of vancomycin (contrary to our results) [19, 55]. This indicates that antibiotics do play a role in TA system activation.

## Conclusions

Our study reveals that activated MazF toxin in the *mazE* mutant strain can lead to increased biofilm formation compared to wildtype. The increased biofilm formation in the *mazE* mutant can be attributed to the increased expression of *cidA* gene which is mediating the eDNA release required for efficient biofilm formation. Although both *mazE* mutant, as well as *mazF* mutant, showed reduced hemolytic activity, murine experiments suggested that *mazEF* TA system does not play any significant role in controlling host–pathogen mediated virulence. The activated MazF toxin in the *mazE* mutant strain confers resistance to antibiotics like oxacillin, daptomycin and vancomycin. Strains resistant to different antibiotic groups have emerged in the past decades. Drugs like vancomycin and daptomycin have long been considered the last resort against infection caused by MRSA. We have come into a generation in which it has been necessary to invent novel targets to which therapeutic approaches can be established. Thus, *mazEF* TA-based antibacterial strategies can be employed for treating infections caused by these resistant superbugs.

## Supplementary Information

**Additional file 1. Table S1**. List of bacterial strains, plasmids, and primers used in this present study; **Table S2**: Transcriptomics data showing a list of significant differentially expressed genes, for wildtype P-1780 vs *mazE* mutant strain, having p-value <=0.05; **Figure S3**. Melting curve analysis of the different genes validated by qPCR; **Figure S4**. Survival curve showing no significant difference in virulence of the generated mutant strains; **Additional materials and methods**.

## Data Availability

All the data sets generated and the transcriptomics data file can be obtained from the corresponding author on reasonable request.

## Abbreviations

MRSA: Methicillin resistant *Staphylococcus aureus*
CRISPR: Clustered Regularly Interspaced Short Palindromic Repeats
Agr: Accessory gene regulator
SSC: Staphylococcal Cassette Chromosome
C: Cytosine
T: Thymine
ESAT-6: Early Secreted Antigenic Target-6 kiloDalton
ATP: Adenosine 5ʹ-triphosphate
OD: Optical density
DEPC: Diethyl pyrocarbonate
CFU: Colony forming units
