# Supplementary material for: Unravelling the physiological roles of mazEF toxin–antitoxin system on clinical MRSA strain by CRISPR RNA-guided cytidine deaminase
Source: J Biomed Sci. 2022 May 7;29:28. doi: 10.1186/s12929-022-00810-5 (PMC9077811; doi:10.1186/s12929-022-00810-5)
Supplement: Supplementary file 1 — Additional file 1. Table S1. List of bacterial strains, plasmids, and primers used in this present study; Table S2: Transcriptomics data showing a list of significant differentially expressed genes, for wildtype P-1780 vs mazE mutant strain, having p-value <=0.05; Figure S3. Melting curve analysis of the different genes validated by qPCR; Figure S4. Survival curve showing no significant difference in virulence of the generated mutant strains; Additional materials and methods. [file 12929_2022_810_MOESM1_ESM.docx]

# Supplementary Information

# Table S1. List of bacterial strains, plasmids, and primers used in this present study

| **Bacterial strains, plasmids, and primers** | **Relevant characteristics** | **Source of reference** | |
| --- | --- | --- | --- |
| **Bacterial strain** | | | |
| Wildtype P-1780 | MRSA ST239 HA strain, *SCC mec* III, *agr* system I, containing exotoxins. isolated from patient post-operative infection, Medical college, India | [13] | |
| RN4220 | Restriction-deficient transformation recipient | ATCC | |
| *E.coli* DH5α | recA1 endA1 gyrA96 thi-1 hsdR17 supE44 relA1 ΔlacU169 (φ80lacZΔM15) | Commercially available | |
| *mazE* mutant | MRSA 1780 *mazE* Q14 mutation to stop codon. | The present study | |
| *mazF* mutant | MRSA 1780 *mazF* Q16 mutation to stop codon. | The present study | |
| *mazE* mutant/pCL55 carrying *mazE* gene | *mazE* mutant complemented with pCL55 carrying *mazE* gene | The present study | |
| *mazF* mutant/pCL55 carrying *mazF* gene | *mazF* mutant complemented with pCL55 carrying *mazF* gene | The present study | |
| RN4220 *mazE* Q14 to stop  RN4220 *mazF* Q16 to stop P1780 ST239 *mazE* Q14 to stop  P1780 ST239 *mazF* Q16 to stop | RN4220 with *mazF* Q14 mutation to stop codon  RN4220 with *mazE* Q16 mutation to stop codon  ST239 with *mazE* Q14 mutation to stop codon  ST239 with *mazF* Q16 mutation to stop codon | The present study | |
| **Plasmids** | | | |
|  | |  | The present study |
| pnCasSA-BEC | *S.aureus* genome editing vector, Kmr, Cmr. (Bacterial expression of Cas9 nuclease, tracrRNA and crRNA guide) |  |  |
| pCasSA-BEC-*mazE* spacer | pCasSA derivative with *mazE* spacer, Q14 mutation to stop codon |  |  |
| pCasSA-BEC-*mazF* spacer | pCasSA derivative with *mazF* spacer, Q16 mutation to stop codon |  |  |
| pCL55 | Single-site integration vector for *S. aureus*.  AmpR in E. coli, CmR in S. aureus |  | [22] |
| pYJ335 | *S. aureus*–*E. coli* shuttle vector containing a tetracycline-inducible promoter |  | The present study |
| **Primers** | | | |
| MazEBaSpF  (Forward *mazE* spacer for its Q 1 4 mutation to stop codon) | 5‘-GAAAttagaacaatctttaaaagaagg-3‘ | The present study | |
| MazEBaSpR  (Reverse *mazE* spacer for its Q 1 4 mutation to stop codon) | 5’-AAACccttcttttaaagattgttctaa -3’ | The present study | |
| mazFBaSpF  (Forward *mazF* spacer for its Q 1 6 mutation to stop  codon) | 5’-GAAAccagtacagggatctgaacaagg-3’ | The present study | |
| mazFBaSpR  (Reverse *mazF*  spacer for its Q 1 6 mutation to stop codon) | 5’-AAACccttgttcagatccctgtactgg-3’ | The present study | |
| mazEvF  (Forward primer for amplification of the verified *mazE* DNA from genome) | 5’-AGAAGGATATTCACAAATGGCTGA-3’, | The present study | |
| mazFvR  (Reverse primer for amplification of the verified *mazF* DNA from genome) | 5’- ATCATCGGATAAGTACGTCAGTTT -3’ | The present study | |
| Forward primer for cloning *lrgB* gene into pYJ335 vector | 5’-ATCAATAGAGGAGGCACAAGCTCATG-3’ | The present study | |
| Reverse primer for cloning *lrgB* gene into pYJ335 vector | 5’-ATCTTAGAAGAATATTGCTACAAAGAC-3’ | The present study | |
| qPCR forward primer for *sarA* gene. | 5’-TCAATGATTGCTTTGAGTTGTT-3’ | The present study | |
| qPCR reverse primer for *sarA* gene. | 5’-TTCTCTTTGTTTTCGCTGATG-3’ | The present study | |
| qPCR forward primer for *saeP* gene. | 5’-AACGGTGAAACTGTTGAAGG-3’ | The present study | |
| qPCR reverse primer for *saeP* gene. | 5’-ACCATTGCGATTTCTTTACC-3’ | The present study | |
| qPCR forward primer for *atl* gene. | 5’-AAGCAATCGTTGGTGGTG-3’ | The present study | |
| qPCR reverse primer for *atl* gene. | 5’-TTGGTGTGTTCCTGGATGTG-3’ | The present study | |
| qPCR forward primer for *mecA* gene. | 5’-AGTTGTAGTTGTCGGGTTT-3’ | The present study | |
| qPCR reverse primer for *mecA* gene. | 5’-AGTGGAACGAAGGTATCATC-3’ | The present study | |
| qPCR forward primer for *femA* gene. | 5’-AGACAAATAGGAGTAATGAT-3’ | The present study | |
| qPCR reverse primer for *femA* gene. | 5’-AAATCTAACACTGAGTGATA-3’ | The present study | |
| qPCR forward primer for *hlgA* gene. | 5’-TCAATCGGAGGCAGTGG-3’ | The present study | |
| qPCR reverse primer for *hlgA* gene. | 5’-CTTGACCATTCGGTGTAACG-3’ | The present study | |
| qPCR forward primer for *hla* gene. | 5’-CGGTATATGGCAATCAACTT-3’ | The present study | |
| qPCR reverse primer for *hla* gene. | 5’-GTCTGGTGAAAACCCTGAAG-3’ | The present study | |
| qPCR forward primer for *cidA* gene. | 5’-CAAACTACTACTACAACTAGGAA-3’ | The present study | |
| qPCR reverse primer for *cidA* gene. | 5’-AGTTAGCGGTACAATCTTAAATT-3’ | The present study | |
| qPCR forward primer for *lrgB* gene. | 5’-TTTCTTAGCGACCATATTATTTG-3’ | The present study | |
| qPCR reverse primer for *lrgB* gene. | 5’-ATAATGTCTCCACCTATTTTGTA-3’ | The present study | |
| qPCR forward primer for *vraS* gene. | 5’-AATCTTTAGGTGAGGGTATTAAA-3’ | The present study | |
| qPCR reverse primer for *vraS* gene. | 5’-GTGTAATTCTGAACAAATGATCT-3’ | The present study | |

**Table S2:** Transcriptomics data showing significant differential expression of different genes, for wildtype P-1780 vs *mazE* mutant strain, having p-value <=0.05. Genes having (-2 ≥ Log_2_[Foldchange] ≥2) are considered differentially expressed.

| **Gene ID** | **Product** | **Gene** | **Log_2_(FoldChange)** | **p-value** |
| --- | --- | --- | --- | --- |
| SAUSA300_0032 | penicillin-binding protein 2' | mecA | 3 | 0.04 |
| SAUSA300_0041 | conserved hypothetical protein | SAUSA300_0041 | -4.2 | 0.02 |
| SAUSA300_0055 | alcohol dehydrogenase%2C zinc-containing | SAUSA300_0055 | -7.2 | 0 |
| SAUSA300_0061 | carbamate kinase | arcC | -4.8 | 0.01 |
| SAUSA300_0072 | hypothetical protein | SAUSA300_0072 | -7.2 | 0.04 |
| SAUSA300_0078 | ATPase copper transport | copA | -7.3 | 0.04 |
| SAUSA300_0113 | immunoglobulin G binding protein A precursor | SAUSA300_0113 | -4.8 | 0 |
| SAUSA300_0131 | putative Bacterial sugar transferase | SAUSA300_0131 | 4 | 0.05 |
| SAUSA300_0132 | glycosyl transferase%2C group 1 family protein | SAUSA300_0132 | 3.5 | 0.04 |
| SAUSA300_0170 | aldehyde dehydrogenase | SAUSA300_0170 | -6.4 | 0 |
| SAUSA300_0179 | putative D-isomer specific 2-hydroxyacid dehydrogenase | SAUSA300_0179 | 6.7 | 0 |
| SAUSA300_0188 | branched-chain amino acid transport system II carrier protein | brnQ | -3.6 | 0.02 |
| SAUSA300_0192 | conserved hypothetical protein | SAUSA300_0192 | -4.9 | 0 |
| SAUSA300_0193 | conserved hypothetical protein | SAUSA300_0193 | -5.8 | 0 |
| SAUSA300_0194 | sucrose-specific PTS tranporter protein | SAUSA300_0194 | -3.6 | 0.02 |
| SAUSA300_0195 | RpiR family transcriptional regulator | SAUSA300_0195 | -3.3 | 0.03 |
| SAUSA300_0199 | conserved hypothetical protein | SAUSA300_0199 | -5.5 | 0 |
| SAUSA300_0206 | flavodoxin family protein | SAUSA300_0206 | 4.7 | 0.01 |
| SAUSA300_0210 | maltose ABC transporter%2C permease protein | SAUSA300_0210 | -3.3 | 0.04 |
| SAUSA300_0211 | maltose ABC transporter%2C permease protein | SAUSA300_0211 | -3.1 | 0.05 |
| SAUSA300_0220 | formate acetyltransferase | pflB | -3.1 | 0.04 |
| SAUSA300_0221 | pyruvate formate-lyase activating enzyme | pflA | -3 | 0.04 |
| SAUSA300_0225 | putative acyl-CoA acetyltransferase FadA | SAUSA300_0225 | -6.5 | 0 |
| SAUSA300_0226 | 3-hydroxyacyl-CoA dehydrogenase | SAUSA300_0226 | -6.3 | 0 |
| SAUSA300_0227 | acyl-CoA dehydrogenase FadD | fadD | -7 | 0 |
| SAUSA300_0228 | acyl-CoA synthetase FadE | fadE | -4.3 | 0.01 |
| SAUSA300_0229 | putative acyl-CoA transferase FadX | SAUSA300_0229 | -5.3 | 0 |
| SAUSA300_0231 | ABC transporter%2C substrate-binding protein | SAUSA300_0231 | 3.4 | 0.03 |
| SAUSA300_0241 | PTS system%2C sorbitol-specific IIC component | SAUSA300_0241 | -3.4 | 0.03 |
| SAUSA300_0242 | sorbitol dehydrogenase | gutB | -3.3 | 0.03 |
| SAUSA300_0244 | oxidoreductase%2C zinc-binding dehydrogenase family | SAUSA300_0244 | -3.5 | 0.03 |
| SAUSA300_0257 | Antiholin-like protein lrgB | SAUSA300_0257 | -3.7 | 0.02 |
| SAUSA300_0278 | conserved hypothetical protein | SAUSA300_0278 | -3.6 | 0.02 |
| SAUSA300_0288 | **Type VII secretion system protein** | essD | -8 | 0.01 |
| SAUSA300_0302 | conserved hypothetical protein | SAUSA300_0302 | -3.2 | 0.05 |
| SAUSA300_0316 | ROK family protein | SAUSA300_0316 | -3.6 | 0.03 |
| SAUSA300_0319 | putative membrane protein | SAUSA300_0319 | -4.9 | 0.01 |
| SAUSA300_0330 | putative transport protein SgaT | SAUSA300_0330 | -3 | 0.05 |
| SAUSA300_0332 | PTS system%2C IIA component | SAUSA300_0332 | -4 | 0.04 |
| SAUSA300_0391 | conserved hypothetical protein | SAUSA300_0391 | 3.6 | 0.03 |
| SAUSA300_0412 |  | SAUSA300_0412 | -3.3 | 0.04 |
| SAUSA300_0424 | putative cobalamin synthesis protein | SAUSA300_0424 | 3.3 | 0.04 |
| SAUSA300_0425 | NADH dehydrogenase I%2C F subunit | SAUSA300_0425 | 3 | 0.05 |
| SAUSA300_0443 | YibE/F-like protein | SAUSA300_0443 | 3.2 | 0.04 |
| SAUSA300_0456 | 23S ribosomal RNA | rrlA | -3.5 | 0.02 |
| SAUSA300_0457 | 5S ribosomal RNA | rrfA | -5 | 0.01 |
| SAUSA300_0463 | conserved hypothetical protein | SAUSA300_0463 | 2.9 | 0.05 |
| SAUSA300_0464 | Methyltransferase | SAUSA300_0464 | 3.1 | 0.05 |
| SAUSA300_0466 | conserved hypothetical protein | SAUSA300_0466 | 3 | 0.05 |
| SAUSA300_0472 | 4-diphosphocytidyl-2C-methyl-D-erythritol kinase | ispE | 3.2 | 0.03 |
| SAUSA300_0497 | 5S ribosomal RNA | rrfG | -4.8 | 0.01 |
| SAUSA300_0501 | 23S ribosomal RNA | rrlB | -3.5 | 0.02 |
| SAUSA300_0502 | 5S ribosomal RNA | rrfB | -5 | 0.01 |
| SAUSA300_0558 | putative proline/betaine transporter | SAUSA300_0558 | 3.1 | 0.04 |
| SAUSA300_0572 | mevalonate kinase | mvk | 3.5 | 0.02 |
| SAUSA300_0574 | phosphomevalonate kinase | SAUSA300_0574 | 2.8 | 0.05 |
| SAUSA300_0575 | conserved hypothetical protein | SAUSA300_0575 | 4.4 | 0.01 |
| SAUSA300_0594 | alcohol dehydrogenase | adh | -3.2 | 0.03 |
| SAUSA300_0605 | staphylococcal accessory regulator A | sarA | 2.4 | 0.05 |
| SAUSA300_0692 | conserved hypothetical protein | SAUSA300_0692 | -4.2 | 0.01 |
| SAUSA300_0693 | putative lipoprotein | SAUSA300_0693 | -3 | 0.05 |
| SAUSA300_0721 | transferrin receptor | SAUSA300_0721 | -3.1 | 0.05 |
| SAUSA300_0773 | putative staphylocoagulase | SAUSA300_0773 | 3.3 | 0.05 |
| SAUSA300_0774 | secretory extracellular matrix and plasma binding protein | empbp | -3.2 | 0.05 |
| SAUSA300_0860 | Ornithine aminotransferase | rocD | -4.5 | 0.01 |
| SAUSA300_0861 | NAD-specific glutamate dehydrogenase | gudB | -3.2 | 0.03 |
| SAUSA300_0862 | glycerophosphoryl diester phosphodiesterase | glpQ | -3.6 | 0.02 |
| SAUSA300_0887 | oligopeptide ABC transporter%2C permease protein | oppB | -4 | 0.01 |
| SAUSA300_0888 | oligopeptide ABC transporter%2C permease protein | oppC | -4.4 | 0.01 |
| SAUSA300_0889 | oligopeptide ABC transporter%2C ATP-binding protein | oppD | -4.5 | 0.01 |
| SAUSA300_0890 | oligopeptide ABC transporter%2C ATP-binding protein | oppF | -4 | 0.01 |
| SAUSA300_0891 | oligopeptide ABC transporter%2C substrate-binding protein | oppA | -4.1 | 0.01 |
| SAUSA300_0929 | conserved hypothetical protein | SAUSA300_0929 | -3.1 | 0.04 |
| SAUSA300_0933 | conserved hypothetical protein | SAUSA300_0933 | -7.6 | 0 |
| SAUSA300_0977 | cobalt transport family protein | SAUSA300_0977 | -3 | 0.04 |
| SAUSA300_0978 | ABC transporter%2C ATP-binding protein | SAUSA300_0978 | -3 | 0.04 |
| SAUSA300_0979 | conserved hypothetical protein | SAUSA300_0979 | -2.8 | 0.05 |
| SAUSA300_1004 | conserved hypothetical protein | SAUSA300_1004 | -3.4 | 0.03 |
| SAUSA300_1008 | conserved hypothetical protein | SAUSA300_1008 | -2.9 | 0.05 |
| SAUSA300_1010 | conserved hypothetical protein | SAUSA300_1010 | -2.9 | 0.05 |
| SAUSA300_1052 | fibrinogen-binding protein | SAUSA300_1052 | -4.2 | 0.01 |
| SAUSA300_1058 | alpha-hemolysin precursor | SAUSA300_1058 | -3.6 | 0.03 |
| SAUSA300_1117 | 50S ribosomal protein L28 | rpmB | -3.2 | 0.04 |
| SAUSA300_1138 | succinyl-CoA synthetase%2C beta subunit | sucC | -3.3 | 0.03 |
| SAUSA300_1139 | succinyl-CoA synthetase%2C alpha subunit | sucD | -3.2 | 0.04 |
| SAUSA300_1313 | carboxyl-terminal protease | ctpA | 2.9 | 0.05 |
| SAUSA300_1314 | conserved hypothetical protein | SAUSA300_1314 | 3 | 0.04 |
| SAUSA300_1316 | methionine-R-sulfoxide reductase | msrB | 2.9 | 0.05 |
| SAUSA300_1325 | conserved hypothetical protein | SAUSA300_1325 | -5 | 0 |
| SAUSA300_1388 | phiSLT ORF488-like protein | SAUSA300_1388 | -3.8 | 0.02 |
| SAUSA300_1424 | conserved hypothetical phage protein | SAUSA300_1424 | -3.3 | 0.05 |
| SAUSA300_1515 | ABC transporter%2C permease protein | SAUSA300_1515 | 3.2 | 0.04 |
| SAUSA300_1516 | ABC transporter%2C ATP-binding protein | SAUSA300_1516 | 4 | 0.01 |
| SAUSA300_1582 | conserved hypothetical protein | SAUSA300_1582 | 2.9 | 0.05 |
| SAUSA300_1627 | translation initiation factor IF-3 | infC | 2.9 | 0.05 |
| SAUSA300_1633 | glyceraldehyde-3-phosphate dehydrogenase%2C type I | gap | -4.9 | 0 |
| SAUSA300_1662 | aminotransferase%2C class V | SAUSA300_1662 | 3.2 | 0.04 |
| SAUSA300_1678 | formate-tetrahydrofolate ligase | fhs | -3.1 | 0.04 |
| SAUSA300_1679 | acetyl-coenzyme A synthetase | acsA | -6.3 | 0 |
| SAUSA300_1680 | acetoin utilization protein AcuA | acuA | -3.6 | 0.02 |
| SAUSA300_1681 | acetoin utilization protein AcuC | acuC | -3.4 | 0.03 |
| SAUSA300_1708 | staphylococcal accessory regulator Rot | rot | -3.5 | 0.02 |
| SAUSA300_1711 | proline dehydrogenase | putA | -7 | 0 |
| SAUSA300_1731 | phosphoenolpyruvate carboxykinase (ATP) | pckA | -5.3 | 0 |
| SAUSA300_1739 | conserved hypothetical protein | SAUSA300_1739 | -4.5 | 0.01 |
| SAUSA300_1740 | conserved hypothetical protein | SAUSA300_1740 | -4.4 | 0.01 |
| SAUSA300_1767 | lantibiotic epidermin biosynthesis protein EpiA | epiA | -3.6 | 0.02 |
| SAUSA300_1773 | tRNA-Glu | SAUSA300_1773 | 4.3 | 0.05 |
| SAUSA300_1778 | tRNA-Asp | SAUSA300_1778 | 3.5 | 0.03 |
| SAUSA300_1801 | fumarate hydratase%2C class II | fumC | -2.9 | 0.05 |
| SAUSA300_1837 | 5S ribosomal RNA | rrfC | -4.9 | 0.01 |
| SAUSA300_1838 | 23S ribosomal RNA | rrlC | -3.5 | 0.02 |
| SAUSA300_1865 | DNA-binding response regulator | vraR | 3.1 | 0.04 |
| SAUSA300_1866 | two-component sensor histidine kinase | vraS | 3.5 | 0.02 |
| SAUSA300_1867 | conserved hypothetical protein | SAUSA300_1867 | 3.3 | 0.03 |
| SAUSA300_1883 | high affinity proline permease | putP | -3 | 0.05 |
| SAUSA300_1922 | staphylokinase precursor | sak | -3.9 | 0.02 |
| SAUSA300_1934 | phi77 ORF020-like protein%2C phage major tail protein | SAUSA300_1934 | -3.4 | 0.03 |
| SAUSA300_1968 | putative phage transcriptional regulator | SAUSA300_1968 | -7 | 0.05 |
| SAUSA300_1969 | phi77 ORF011-like protein%2C phage transcriptional repressor | SAUSA300_1969 | -4.4 | 0.05 |
| SAUSA300_1981 | phage terminase family protein | SAUSA300_1981 | 3.6 | 0.02 |
| SAUSA300_1986 | nitroreductase family protein | SAUSA300_1986 | 4.1 | 0.01 |
| SAUSA300_2015 | 5S ribosomal RNA | rrfD | -4.9 | 0.01 |
| SAUSA300_2016 | 23S ribosomal RNA | rrlD | -3.5 | 0.02 |
| SAUSA300_2037 | ATP-dependent RNA helicase | cshA | 4.5 | 0.01 |
| SAUSA300_2048 | hydroxyethylthiazole kinase | thiM | -3 | 0.04 |
| SAUSA300_2092 | general stress protein 20U | dps | 6 | 0 |
| SAUSA300_2104 | glucosamine--fructose-6-phosphate aminotransferase (isomerizing) | glmS | -3 | 0.04 |
| SAUSA300_2122 | 5S ribosomal RNA | rrfE | -4.9 | 0.01 |
| SAUSA300_2123 | 23S ribosomal RNA | rrlE | -3.5 | 0.02 |
| SAUSA300_2132 | conserved hypothetical protein | SAUSA300_2132 | -3.6 | 0.02 |
| SAUSA300_2165 | alpha-acetolactate decarboxylase | budA | 6.3 | 0 |
| SAUSA300_2166 | alpha-acetolactate synthase | alsS | 6.9 | 0 |
| SAUSA300_2237 | putative urea transporter | SAUSA300_2237 | 4.3 | 0.01 |
| SAUSA300_2239 | urease%2C beta subunit | ureB | 7.6 | 0 |
| SAUSA300_2240 | urease%2C alpha subunit | ureC | 6.1 | 0 |
| SAUSA300_2241 | urease accessory protein UreE | ureE | 4.9 | 0 |
| SAUSA300_2242 | urease accessory protein UreF | ureF | 5.2 | 0 |
| SAUSA300_2243 | urease accessory protein UreG | ureG | 4.3 | 0.01 |
| SAUSA300_2244 | urease accessory protein UreD | ureD | 4.8 | 0.01 |
| SAUSA300_2274 | putative membrane protein | SAUSA300_2274 | -3 | 0.05 |
| SAUSA300_2277 | imidazolonepropionase | hutI | -3.3 | 0.03 |
| SAUSA300_2278 | urocanate hydratase | hutU | -4.2 | 0.01 |
| SAUSA300_2299 | multidrug resistance protein A%2C drug resistance transporter | SAUSA300_2299 | 5.5 | 0 |
| SAUSA300_2302 | teicoplanin resistance associated membrane protein TcaA protein | tcaA | 3.2 | 0.03 |
| SAUSA300_2310 | conserved hypothetical protein | SAUSA300_2310 | -2.8 | 0.05 |
| SAUSA300_2321 | putative membrane protein | SAUSA300_2321 | 3 | 0.05 |
| SAUSA300_2333 | nitrite extrusion protein | narK | -3.2 | 0.04 |
| SAUSA300_2343 | respiratory nitrate reductase%2C alpha subunit | SAUSA300_2343 | -3.4 | 0.03 |
| SAUSA300_2351 | Zn-binding lipoprotein adcA-like protein | SAUSA300_2351 | 3.4 | 0.03 |
| SAUSA300_2362 | 2%2C3-bisphosphoglycerate-dependent phosphoglycerate mutase | gpmA | 3.8 | 0.02 |
| SAUSA300_2365 | gamma-hemolysin component A | hlgA | -3.6 | 0.03 |
| SAUSA300_2378 | conserved hypothetical protein | SAUSA300_2378 | 2.8 | 0.05 |
| SAUSA300_2383 | amino acid permease | SAUSA300_2383 | -5.5 | 0 |
| SAUSA300_2414 | conserved hypothetical protein | SAUSA300_2414 | 3.6 | 0.03 |
| SAUSA300_2417 | putative transporter | SAUSA300_2417 | -3.4 | 0.03 |
| SAUSA300_2423 | conserved hypothetical protein | SAUSA300_2423 | -3.6 | 0.02 |
| SAUSA300_2448 | putative membrane protein | SAUSA300_2448 | 4.1 | 0.01 |
| SAUSA300_2449 | putative transporter | SAUSA300_2449 | -5.2 | 0 |
| SAUSA300_2453 | ABC transporter%2C ATP-binding protein | SAUSA300_2453 | -5.9 | 0 |
| SAUSA300_2454 | membrane spanning protein | SAUSA300_2454 | -5 | 0.01 |
| SAUSA300_2455 | putative fructose-1%2C6-bisphosphatase | SAUSA300_2455 | -2.9 | 0.05 |
| SAUSA300_2479 | Holin-like protein cidA | cidA | 8.3 | 0 |
| SAUSA300_2481 | conserved hypothetical protein | SAUSA300_2481 | -3.6 | 0.02 |
| SAUSA300_2487 | ferrous iron transport protein B | feoB | 3.5 | 0.03 |
| SAUSA300_2488 | ferrous iron transport protein A | feoA | 5.8 | 0.01 |
| SAUSA300_2491 | 1-pyrroline-5-carboxylate dehydrogenase | SAUSA300_2491 | -5.3 | 0 |
| SAUSA300_2510 | conserved hypothetical protein | SAUSA300_2510 | 3.7 | 0.02 |
| SAUSA300_2511 | conserved hypothetical protein | SAUSA300_2511 | 3.1 | 0.05 |
| SAUSA300_2521 | conserved hypothetical protein | SAUSA300_2521 | 3.9 | 0.03 |
| SAUSA300_2537 | L-lactate dehydrogenase | SAUSA300_2537 | -3.8 | 0.02 |
| SAUSA300_2546 | glycine betaine aldehyde dehydrogenase | betB | 3.8 | 0.02 |
| SAUSA300_2549 | choline/carnitine/betaine transporter%2C BCCT family | bccT | 2.9 | 0.05 |
| SAUSA300_2603 | triacylglycerol lipase precursor | lip | -5.6 | 0 |
| SAUSA300_2620 | conserved hypothetical protein | SAUSA300_2620 | 3.8 | 0.02 |
| SAUSA300_2622 | conserved hypothetical protein | SAUSA300_2622 | 2.9 | 0.05 |
| SAUSA300_2624 | putative membrane protein | SAUSA300_2624 | -7.8 | 0.02 |
| SAUSA300_2639 | cold shock protein | SAUSA300_2639 | 3.5 | 0.02 |

**Figure S3.** Melting curve analysis of the different genes validated by qPCR.

**
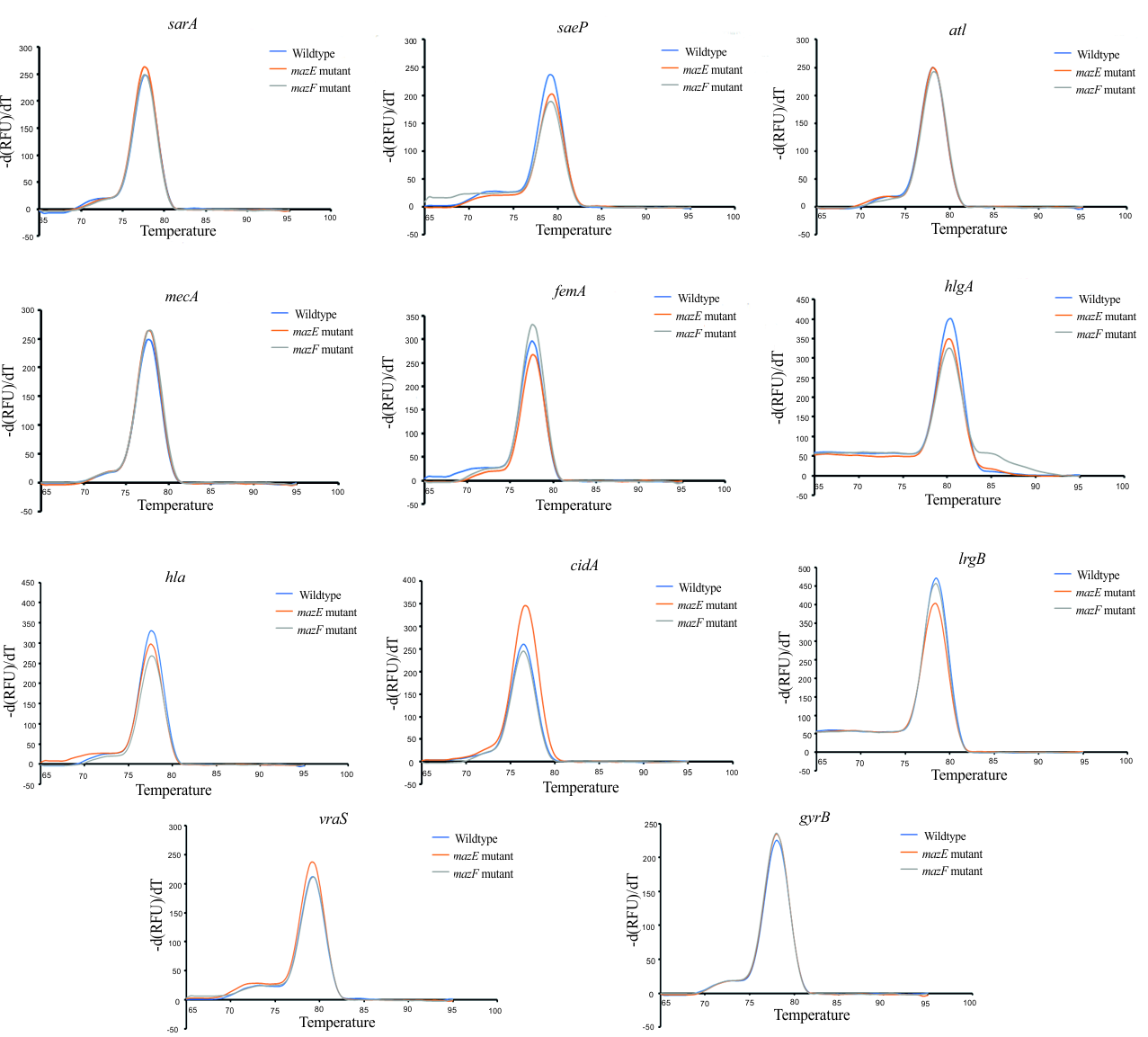
**

**Figure S4.** Survival curve showing no significant difference in virulence of the generated mutant strains

**
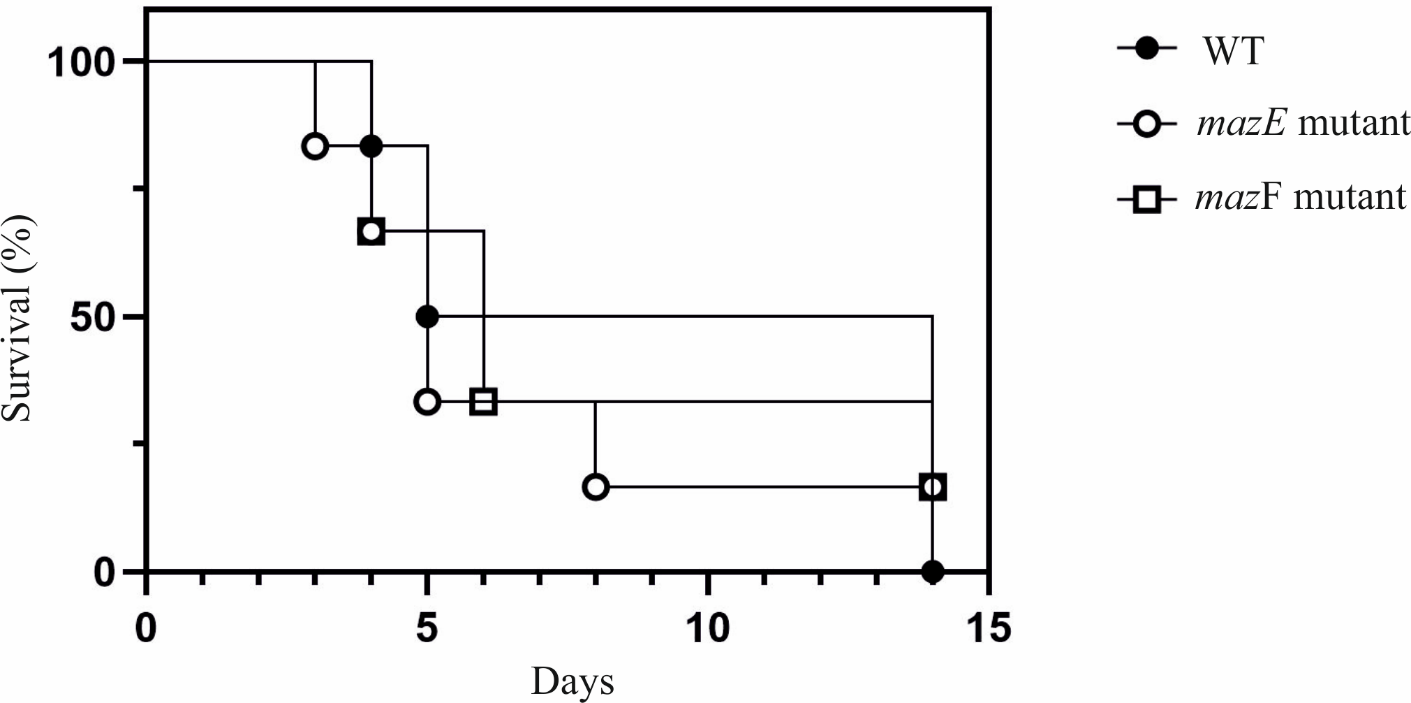
**

**Materials and methods (additional):**

**1.Preparation of electrocompetent *Staphylococcus aureus***

Overnight culture of RN4220 was transferred (1/100 dilution) to 50 ml TSB in 250 ml flask and incubated at 30°C until OD_600_ reaches near 0.5 (in 2.5 – 3 hrs). Cells were harvested by centrifugation at 8000 rpm for 10 mins. The pelleted cells were washed with 50 ml of sterile ice-cold 0.5M Sucrose (wash solution) followed by again 3 times with 30 ml of ice-cold wash solution. Finally, the cells were resuspended in 0.5 ml of ice-cold wash solution (100 times concentrated). 100µl of cells were aliquoted followed by quick freeze in liquid nitrogen and stored at -80°C. For electroporation: The electroporator was set to 100 Ω, 2.5 kV and 25 µF. Single pulse was given using 0.1 cm cuvette with immediate addition of 1 ml TSB containing chloramphenicol (5µg/ml). The cells were then incubated for 1.5 hrs at 30°C and plated on a TSB agar plate containing 5 μg/mL chloramphenicol. The plate was further incubated at 30°C for 24 h.

**2.Transduction process**

1. **Transducing phage preparation**

Overnight culture of RN4220 carrying pnCasSA-BEC-*mazE*/*mazF*sp plasmid with desired mutation was diluted 1:100 with fresh heart infusion broth (HIB) containing 5 µg/ml chloramphenicol and 5 mM CaCl_2_ and was incubated at 30°C for 2.5-3 h until (OD_600_ = 0.5). 100µl phi85 phage solution (10^10^ pfu/ml) was added into the culture to ensure appropriate transduction frequency (around 10^-8^). Initially, the culture was kept in 30°C shaking for 3 hrs. Then, it was incubated overnight, (without shaking) at 30°C until cells are lysed and appeared clear. It was centrifuged at 14k rpm for 10 mins, followed by filter sterilization with 0.22 µm filter.

1. **Transduction**

Pelleted cells of overnight culture wildtype ST239 (P-1780) was resuspended in the same volume of HIB containing 5 mM CaCl_2_. It was aliquoted into 300 µl eppendorf along with 500 μl of transducing phage solution. A “no-phage” control was prepared simultaneously. The mixture was incubated at 30℃ for 20 min with shaking at 250 rpm.  The cells were spin down at room temperature at 14k rpm, 1 min. Cell pellet was then washed with 1 ml of ice-cold 40 mM sodium citrate and the cells were again spin down at 14k rpm, 1 min. Pellet was resuspended into different aliquots of 100 - 500 µl of ice cold 40 mM sodium citrate and 100 ul from each of the aliquot was plated on TSA with 10 μg/mL chloramphenicol and incubated at 30°C for 2 days. 2-4 colonies which appeared were streaked on TSA plate with 10 µg/ml chloramphenicol. If the streak showed plaques at the beginning of the streak, it confirms that the cells are *S. aureus* colony. The latter part of the streak was then used for inoculation.
